# Identification of rheumatoid arthritis and osteoarthritis patients by transcriptome-based rule set generation

**DOI:** 10.1186/ar4526

**Published:** 2014-04-01

**Authors:** Dirk Woetzel, Rene Huber, Peter Kupfer, Dirk Pohlers, Michael Pfaff, Dominik Driesch, Thomas Häupl, Dirk Koczan, Peter Stiehl, Reinhard Guthke, Raimund W Kinne

**Affiliations:** 1 BioControl Jena GmbH, Wildenbruchstraße 15, 07745 Jena, Germany; 2 Experimental Rheumatology Unit, Department of Orthopedics, Jena University Hospital, Waldkrankenhaus Rudolf Elle, Klosterlausnitzer Straße 81, 07607 Eisenberg, Germany; 3 Institute of Clinical Chemistry, Hannover Medical School, Carl-Neuberg-Straße 1, 30625 Hannover, Germany; 4 Leibniz Institute for Natural Product Research and Infection Biology, Hans Knöll Institute, Beutenbergstraße 11a, 07745 Jena, Germany; 5 Present address: Center of Diagnostics GmbH, Chemnitz Hospital, Flemmingstr. 2, 09116 Chemnitz, Germany; 6 Department of Medical Engineering and Biotechnology, University of Applied Sciences Jena, Carl-Zeiss-Promenade 2, 07745 Jena, Germany; 7 Department of Rheumatology and Clinical Immunology, Charite-Universitätsmedizin Berlin, Chariteplatz 1, 10117 Berlin, Germany; 8 Institute of Immunology, University of Rostock, Schillingallee 68, 18057 Rostock, Germany; 9 Institute of Pathology, University of Leipzig, Liebigstraße 24, 04103 Leipzig, Germany

## Abstract

**Introduction:**

Discrimination of rheumatoid arthritis (RA) patients from patients with other inflammatory or degenerative joint diseases or healthy individuals purely on the basis of genes differentially expressed in high-throughput data has proven very difficult. Thus, the present study sought to achieve such discrimination by employing a novel unbiased approach using rule-based classifiers.

**Methods:**

Three multi-center genome-wide transcriptomic data sets (Affymetrix HG-U133 A/B) from a total of 79 individuals, including 20 healthy controls (control group - CG), as well as 26 osteoarthritis (OA) and 33 RA patients, were used to infer rule-based classifiers to discriminate the disease groups. The rules were ranked with respect to Kiendl’s statistical relevance index, and the resulting rule set was optimized by pruning. The rule sets were inferred separately from data of one of three centers and applied to the two remaining centers for validation. All rules from the optimized rule sets of all centers were used to analyze their biological relevance applying the software Pathway Studio.

**Results:**

The optimized rule sets for the three centers contained a total of 29, 20, and 8 rules (including 10, 8, and 4 rules for ‘RA’), respectively. The mean sensitivity for the prediction of RA based on six center-to-center tests was 96% (range 90% to 100%), that for OA 86% (range 40% to 100%). The mean specificity for RA prediction was 94% (range 80% to 100%), that for OA 96% (range 83.3% to 100%). The average overall accuracy of the three different rule-based classifiers was 91% (range 80% to 100%). Unbiased analyses by Pathway Studio of the gene sets obtained by discrimination of RA from OA and CG with rule-based classifiers resulted in the identification of the pathogenetically and/or therapeutically relevant interferon-gamma and GM-CSF pathways.

**Conclusion:**

First-time application of rule-based classifiers for the discrimination of RA resulted in high performance, with means for all assessment parameters close to or higher than 90%. In addition, this unbiased, new approach resulted in the identification not only of pathways known to be critical to RA, but also of novel molecules such as serine/threonine kinase 10.

## Introduction

Rheumatoid arthritis (RA) and osteoarthritis (OA) are the most common forms of arthritis [1]. In spite of different pathogeneses, these arthritides exhibit phenotypic similarities and overlapping cellular and molecular characteristics [1,2]. RA is a progressive, chronically inflammatory, destructive joint disease of still unknown etiology, perpetuated by an invasive synovial membrane (also known as pannus tissue) [3]. Various activated or semi-transformed cell types in the synovial membrane (monocytes/macrophages, osteoclasts, T cells and B cells, dendritic cells and endothelial cells, synovial fibroblasts) contribute to the development and progression of RA by secretion of proinflammatory cytokines and tissue-degrading proteases [4,5]. Similarly, OA is characterized by progressive destruction of cartilage and bone and dysregulation of synovial function [6]. OA arises from the damage of articular cartilage induced by physical injury and is subsequently influenced by a variety of intrinsic (for example, genetic, cellular, or immunologic) factors [7]. The OA synovial membrane also shows an inflammatory component, although clearly less pronounced than in RA [2,7].

Compatible with these similarities, the synovial tissue of OA and RA patients contains mesenchymal precursor cells and attempts to regenerate damaged cartilage and subchondral bone in the adult organism. In contrast to fetal healing, however, the synovial tissue may require inflammation to sustain and control the fibroproliferation [8].

Although these overlapping features have led to the development of pharmacological or surgical therapies effective in both diseases [9-12], the similarities at the same time impede a reliable discrimination of the two arthritides. Diagnostic methods classically include radiography [13], histopathological assessment of synovitis [14], detection of rheumatic nodules, selected laboratory values such as rheumatoid factor and citrullinated peptides [15,16], and evaluation of the patients’ individual and family history [17]. Recently, an improved ultrasound-based scoring system has also been proposed [18]. In general, American College of Rheumatology criteria for RA [15,19] or for OA [16] are often used for diagnostic purposes, although they were originally intended as classification criteria, for example, for the comparison of cohorts in different clinical studies [20]. However, an appropriate discrimination of RA and OA is particularly difficult at later stages of the diseases, and the recent revision of the respective criteria has not significantly improved their diagnostic capability [20]. For instance, the presence of rheumatoid factor as a marker for RA has been questioned due to its high variability and should be replaced by the level of anti-citrullinated protein antibodies [21].

An easier discrimination of different forms of arthritis has been attempted by molecular approaches, in particular, disease-specific gene expression profiling. These attempts have partially focused on the expression of selected candidate molecules with a known influence on the respective diseases; for example, type I interferon family members [22,23], tumor necrosis factor superfamily and bone morphogenetic protein family members [24], citrullinated synovial proteins [25], and proteases such as metalloproteinases or cathepsins [26]. Although these studies have indicated the existence of individual or combined biomarkers for RA, the validity of this approach has not been universal. Some of the studies have succeeded in discriminating RA from normal controls, but not from other arthritides, while other studies have successfully discriminated RA from other forms of arthritis (such as spondylopathy or psioriatic arthritis), but not from OA [24].

In parallel to candidate gene analyses, broader, unbiased genome-wide gene expression profiles [27] have been used to identify disease-specific signatures and hidden biomarkers in rheumatology with microarray-based methods [28]. This has been applied to discriminate early versus late RA [29] and to discriminate RA versus OA [30,31]. In addition, differentially expressed genes have been successfully used to predict the response of RA patients to therapeutic approaches, for example, the capability of certain (type I interferon-responsive) genes to predict rituximab nonresponders [32] and anti-tumor necrosis factor nonresponders [33] or to define homogeneous subgroups within a heterogeneous disease such as RA [22]. However, most studies were not designed to identify gene expression patterns as a potential diagnostic tool, but rather to elucidate the underlying transcriptional networks [34]. The validity of the identified genes as markers for RA or OA was generally also not validated in replication cohorts. Finally, differentially expressed respectively regulated genes or pathways common to RA and OA remain a major challenge [30].

These obstacles may be overcome using microarray data from several analytic centers to identify sets of differentially expressed genes for the reliable diagnosis of different arthritides. For this purpose, bioinformatic methods suitable to process and interpret the large amounts of high-dimensional data, and also algorithms for the identification of rules concerning the expression of disease-specific genes, are of utmost importance [35].

In personalized medicine and theranostics, the generation of decision rules is a well-established method for the design of clinical decision support systems and/or for the discovery of relevant relationships among pathogenetically relevant genes in large databases [36,37]. This approach is intended to identify strong rules using different measures of so-called interestingness, for example, specificity for a certain disease entity. To select interesting rules from the set of all possible rules, constraints on various measures of significance can be used, such as thresholds on support and confidence. In our hands [38], the relevance index introduced by Kiendl and coworkers [39-43] is able to generate robust rule sets with high predictive strength from data of high dimension (for example, number of genes) but of low sample number. A deterministic decision rule *R*_*r*_ is defined by ‘IF *P*_*r*_(*y*) THEN *C*_*r*_’, where *P*_*r*_ describes a premise evaluating the observations *y* (that is, the enhanced expression of a given gene) and *C*_*r*_ is the set of possible conclusions (for example, the prediction of a disease status of a given individual). In the present work, *C*_*r*_ is a categorical variable defined by the set of three clinical states {‘CG’ – control group, ‘RA’ – rheumatoid arthritis, ‘OA’ – osteoarthritis} and each premise *P*_*r*_ is defined by the expression of only one gene (uniconditional rules).

This rule-oriented approach may represent a more suitable alternative to the widely used identification of differentially expressed genes to generate a sorted list of candidate genes of interest. The approach thus combines three major advantages: i) by avoiding the application of differentially expressed genes, it is more robust in its discriminative capacity to data heterogeneity among different donors or patients; ii) due to separate normalization and independent rule set generation, it is capable of eliminating center-specific effects, thus yielding higher sample sizes in study cohorts; and iii) cross-validation among different clinical centers is possible, independently of individual differentially expressed genes.

In this study, three multicenter genome-wide transcriptomic datasets from 79 individuals were used to infer rule-based classifiers to discriminate RA, OA, and healthy controls. The rule sets were inferred separately from one center and were applied to the other centers for validation. This novel approach resulted in high performance (close to 90% for specificity, sensitivity, and accuracy) for the discrimination of RA. Unbiased analysis of the biological relevance of the underlying rules by Pathway Studio (Elsevier, Munich, Germany) and gene enrichment analysis succeeded in identifying pathways with pathogenetic or therapeutic relevance in RA.

## Materials and methods

### Patients

Synovial membrane samples were obtained either from postmortem joints/traumatic joint injury cases (control group (CG); *n* = 15 and *n* = 5, respectively) or from RA/OA patients (all Caucasian) upon joint replacement/synovectomy at the Jena University Hospital, Chair of Orthopedics, Waldkrankenhaus ‘Rudolf Elle’, Eisenberg, Germany (*n* = 33, dataset ‘Jena’), at the Department of Orthopedics/Institute of Pathology/Department of Rheumatology and Clinical Immunology, Charité-Universitätsmedizin Berlin (*n* = 30, dataset ‘Berlin’), and at the Department of Orthopedics/Institute of Pathology, University of Leipzig (*n* = 16, dataset ‘Leipzig’). After removal, tissue samples were frozen and stored at −70°C.

The study was approved by the respective ethics committees (Jena University Hospital: Ethics Committee of the Friedrich Schiller University Jena at the Medical Faculty; Charité-Universitätsmedizin Berlin: Charité Ethics Committee; and University of Leipzig: Ethics Committee at the Medical Faculty of the University of Leipzig) and informed patient consent was obtained. RA patients were classified according to the American College of Rheumatology criteria valid in the sample assessment period [15], OA patients were classified according to the respective criteria for OA [16]. The patients/donors were assigned to one of the three terms (categorical values): ‘CG’, ‘RA’, or ‘OA’ (for clinical characteristics of the donors/patients, see Table 1).

**Table 1:** Clinical characteristics of the patients at the time of synovectomy/sampling

**Patients (total number)**	**Gender (male/female)**	**Age (Years)**	**Disease duration (years)**	**RF (+/−)**	**ESR (mm/1 hour)**	**CRP**^**a **^**(mg/l)**	**Number of ARA-criteria (RA)**	**Concomitant medication (*n *)**
Control group								
(*n* = 20)	15/5	54.7 ± 4.0	0.3 ± 0.3^b^	n.d.	n.d.	n.d.	n.a.	NSAIDs (*n* = 1)
			(n.d. = 13)					None (*n* = 7)
								(n.d. = 12)
Osteoarthritis								
(*n* = 26)	4/22	71.0 ± 1.4	7.0 ± 1.3	3/18	22.4 ± 2.7	5.3 ± 1.5	0.2 ± 0.1	NSAIDs (*n* = 16)
			(n.d. = 1)	(n.d. = 5)	(n.d. = 5)	(n.d. = 3)		None (*n* = 10)
Rheumatoid arthritis								
(*n* = 33)	8/25	57.0 ± 2.7	12.5 ± 2.0	21/7	42.7 ± 4.5	21.4 ± 4.1	5.2 ± 0.3	Prednisolone (*n* = 23)
				(n.d. = 7)	(n.d. = 10)	(n.d. = 3)		Methotrexate (*n* = 18)
								Sulfasalazine (*n* = 5)
								Chloroquine (*n* = 2)
								Leflunomide (*n* = 2)
								Cyclosporine (*n* = 1)
								Gold (*n* = 1)
								NSAIDs (*n* = 22)

For the parameters age, disease duration, ESR, CRP, and number of ARA criteria (RA), means ± standard error of the mean are given; for the remaining parameters, numbers are provided. ARA, American Rheumatism Association (now American College of Rheumatology); n.a., not applicable; n.d., not determined; NSAID, nonsteroidal anti-inflammatory drug; RA, rheumatoid arthritis; RF, rheumatoid factor; ESR, erythrocyte sedimentation rate; CRP, C-reactive protein.

^a^Normal range, <5 mg /l.

^b^Disease duration in joint trauma patients.

### Data

Data for 79 patients/donors were obtained from three clinical groups located in Jena, Berlin, and Leipzig, respectively, as presented in Table 2.

**Table 2:** Number of clinical samples and transcriptome datasets

**Study group *S***	**Control**	**Osteoarthritis**	**Rheumatoid arthritis**	**Total**	**Microarray platform**^**a**^
‘Jena’	10	10	13	33	Affymetrix HG-U133 A
‘Berlin’	10	10	10	30	Affymetrix HG-U133 A
‘Leipzig’	0	6	10	16	Affymetrix HG-U133 A
‘Total’	20	26	33	79	

^a^From Affymetrix, Santa Clara, CA, USA.

### Isolation of total RNA

Tissue homogenization, total RNA isolation, and treatment with RNase-free DNase I (Qiagen, Hilden, Germany) were performed as described previously [44].

### Microarray analysis

Gene expression was analyzed using HG-U133 A/B RNA microarrays (Affymetrix, Santa Clara, CA, USA) for the datasets ‘Jena’, ‘Berlin’, and ‘Leipzig’ – a total of 79 microarrays. Labeling of RNA probes, hybridization, and washing were carried out according to the supplier’s instructions. Microarrays were analyzed by laser scanning (Gene Scanner; Hewlett-Packard, Palo Alto, CA, USA).

### Pre-processing of microarray data

Gene expression data were pre-processed by MAS5.0 (Affymetrix Microarray Suite). The data are accessible through Gene Expression Omnibus series [GSE:55235] (Haeupl; ‘Berlin’ data), [GSE:55584] (Stiehl; ‘Leipzig’ data), and [GSE:55457] (Kinne; ‘Jena’ data).

For the study group ‘Jena_all’, all probe sets independent of their Affymetrix ‘present call’ were used for further analysis. For the study groups ‘Jena’, ‘Berlin’, ‘Leipzig’, and ‘Total’, further analyses were restricted to those genes qualified by a ‘present call’ in all samples of the respective study group (as calculated by MAS 5.0). The data were separately normalized for the three different study groups ‘Jena’, ‘Berlin’, and ‘Leipzig’ by dividing the gene expression signals for a given gene *i* and sample/patient *j* by the median over all probe sets in this sample and were subsequently logarithmized (log_2_), yielding the values *y*_*ij*_. By performing completely independent normalization and rule set generation (see Rule set generation) in the three different clinical datasets, potential problems related to differences in sample preparation and wet laboratory conditions were avoided [45].

### Clustering

The data were separately clustered for each probe set (gene) using a modified fuzzy C-means algorithm and two clusters. Here, the fuzzy C-means algorithm [46] was applied for the normalized and logarithmized (log_2_) gene expression data (*y*_*ij*_) of a given gene for every patient belonging to the respective group (that is, ‘Jena_all’, ‘Jena’, ‘Berlin’, ‘Leipzig’, or ‘Total’) to estimate membership degrees (*M*_*ijk*_) ranging from 0 to 1 for unequivocal assignment to one of the groups ‘low’ or ‘high’ gene expression. The centers (*CT*_*ik*_; *CT*_*i*1_ < *CT*_*i*2_) of the respective gene expression clusters (*CL*_*ik*_, *k* = 1 for the cluster labeled ‘low’ and *k* = 2 for that labeled ‘high’) were also estimated. Subsequently, a modified membership degree was used (*M*_*ijk*_*′;* with *M*_*ij*1_*′* = 1 and *M*_*ij*2_*′* = 0 if *y*_*ij*_ < *CT*_*i*1_; with *M*_*ij*1_*′* = 0 and *M*_*ij*2_*′* = 1 if *y*_*ij*_ > *CT*_*i*2_; with *M*_*ijk*_*′* = *M*_*ijk*_ otherwise; that is, for all data in between the two centers).

### Rule set generation

First, all uniconditional rules were generated independently for the three different clinical study groups ‘Jena’, ‘Berlin’, and ‘Leipzig’ using the formula ‘IF the premise *P*_*r*_ is fulfilled THEN the conclusion *C*_*r*_ is reached’*.* The premise *P*_*r*_ is defined as follows: the expression of gene *i* belongs to either the cluster labeled ‘low’ (*CL*_*i*1_) or the cluster labeled ‘high’ (*CL*_*i*2_)*.* The three possible conclusions (*C*_*r*_; that is, in the present study the prediction of the clinical status) are ‘CG’ (that is, no ‘RA’, no ‘OA’), ‘RA’, or ‘OA’.

These rules were ranked using the relevance index *RI*_*r*_ introduced by Kiendl and others [39-43]. Here, a rule ‘IF *P*_*r*_ THEN *C*_*r*_’ is ranked on the basis of *RI*_*r*_. In this case, *RI*_*r*_ represents the normalized gap between the confidence interval of the conditional probability of the conclusion *C*_*r*_ under the premise *P*_*r*_ and the confidence interval of the (unconditional) probability of the conclusion *C*_*r*_, as described in Additional file 1. The calculation of the confidence interval was done using a significance level *alpha*_*S*_ with a default value 0.95, and a reduced *alpha*_*S*_ for ‘Jena’, ‘Berlin’, and ‘Leipzig’ in order to generate a sufficient number (>3) of rules with *RI*_*r*_ > 0 for each conclusion (‘CG’, ‘RA’, or ‘OA’). Next, it was checked and confirmed that *alpha*_*S*_ > *alpha*_*S*random_, where at least one rule was generated for each of the three conclusions using original pre-processed gene expression values *y*_*ij*_, and a random assignment to the individual conclusions (‘CG’, ‘RA’, and ‘OA’) in the training set.

### Rule set pruning

As a result of the primary rule set generation, a ranked set of *r*_*max*_(*C*, *S*) rules was generated using the criterion *RI*_*r*_ > 0.

Rule set pruning was then applied in order to minimize the numbers of both rules (*r*_*opt*_) and ‘Errors’ (that is, false assignment to one of the three conclusions; for more detail see Application of the rule sets and Evaluation of a rule set). The number of rules in each rule set was optimized by greedy search with the following constraints: the numbers *r*_*opt*_(C, S) have to be at least 4 for each conclusion and not higher than the double of the minimum number of rules in any of the respective rule sets for the three conclusions – that is, *r*_*opt*_(*C*, *S*) ≥ 4 and *r*_*opt*_(*C*, *S*) ≤ 2* min_C_(*r*_*max*_(*C*, *S*)).

The purpose of this step was also to generate rule sets with a balanced number of rules for the three conclusions.

### Application of the rule sets

The rule sets for the different conclusions were then applied to each sample (patient) *j* by voting in order to achieve an individual prediction of its clinical status.

**Figure 1:**
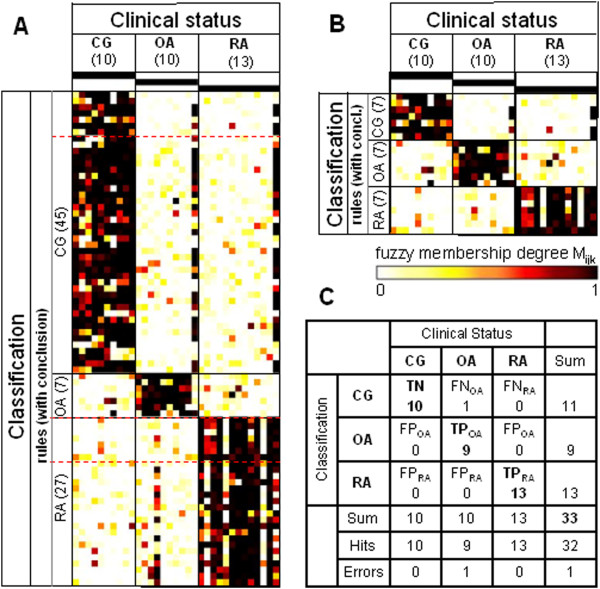
**Heatmaps and confusion matrix for the study group ‘Jena_all’.** Data for the study group ‘Jena_all’ (that is, utilizing all probe sets) were obtained using the Jena patients (10 control group (CG), 10 osteoarthritis (OA), 13 rheumatoid arthritis (RA)) as the training set for the rule generation and re-applying the respective rules to the same dataset. **(A)** Heatmap of the membership weights applying all rules of the primary rule set (α = 0.95; ‘CG’, 45 rules; ‘OA’, seven rules; ‘RA’, 27 rules) for the prediction of the clinical status of the different samples; dashed red lines indicate the lower limits of the respective pruned lists of rules subsequently applied in **(B)**. **(B)** Heatmap of the membership weights applying pruned lists of rules (α = 0.95; ‘CG’, ‘OA’, and ‘RA’, seven top-ranked rules each) for optimized prediction of the clinical status of the different samples. **(C)** Confusion matrix for the rule set displayed in heatmap **(B)**. TP, true positives; TN, true negatives; FP, false positives; FN, false negatives.

**Figure 2:**
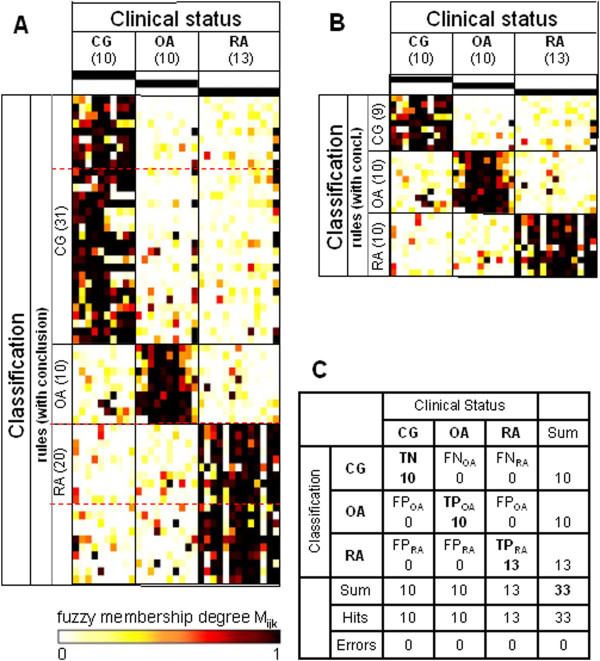
**Heatmaps and confusion matrix for the study group ‘Jena’.** Data for the study group ‘Jena’ (that is, utilizing only the probe sets with MAS 5.0 present calls in all samples) were obtained using the Jena patients (10 control group (CG), 10 osteoarthritis (OA), 13 rheumatoid arthritis (RA)) as the training set for the rule generation and re-applying the respective rules to the same dataset. **(A)** Heatmap of the membership weights applying all rules of the primary rule set (α = 0.94; ‘CG’, 31 rules; ‘OA’, 10 rules; ‘RA’, 20 rules) for the prediction of the clinical status of the different samples; dashed red lines indicate the lower limits of the respective pruned lists of rules subsequently applied in **(B)**. **(B)** Heatmap of the membership weights applying pruned lists of rules (α = 0.94; ‘CG’, nine top-ranked rules; ‘OA’, 10 top-ranked rules; ‘RA’, 10 top-ranked rules) for optimized prediction of the clinical status of the different samples. **(C)** Confusion matrix for the rule set displayed in heatmap **(B)**. TP, true positives; TN, true negatives; FP, false positives; FN, false negatives.

**Figure 3:**
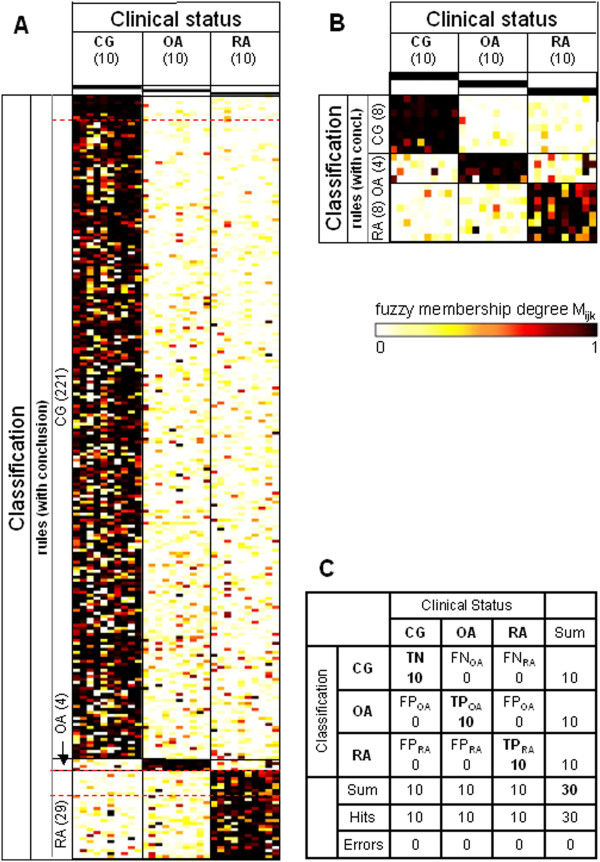
**Heatmaps and confusion matrix for the study group ‘Berlin’.** Data were obtained using the study group ‘Berlin’ (10 control group (CG), 10 osteoarthritis (OA), 10 rheumatoid arthritis (RA)) as the training set for the rule generation and re-applying the respective rules to the same dataset. **(A)** Heatmap of the membership weights applying all rules of the primary rule set (α = 0.94; ‘CG’, 221 rules; ‘OA’, four rules; ‘RA’, 29 rules) for the prediction of the clinical status of the different samples; dashed red lines indicate the lower limits of the respective pruned lists of rules subsequently applied in **(B)**. **(B)** Heatmap of the membership weights applying pruned lists of rules (α = 0.94; ‘CG’, eight top-ranked rules; ‘OA’, four top-ranked rules; ‘RA’, eight top-ranked rules) for optimized prediction of the clinical status of the different samples. **(C)** Confusion matrix for the rule set displayed in heatmap **(B)**. TP, true positives; TN, true negatives; FP, false positives; FN, false negatives.

**Figure 4:**
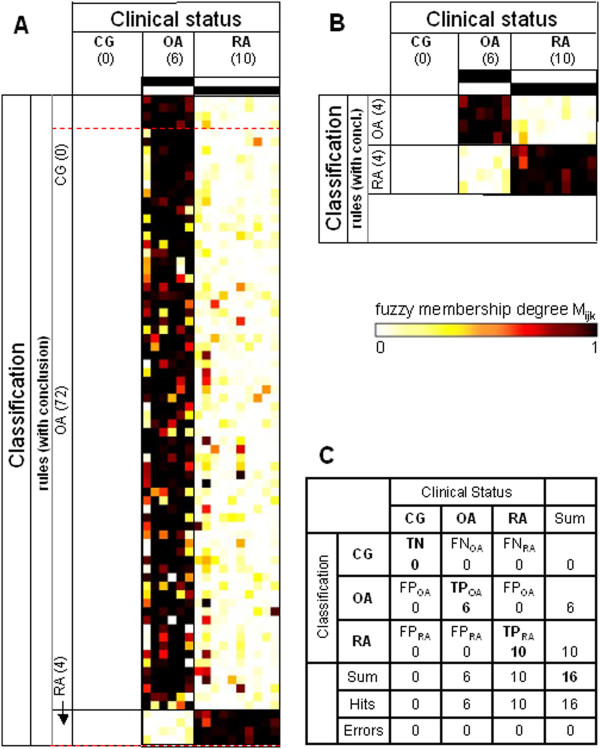
**Heatmaps and confusion matrix for the study group ‘Leipzig’.** Data were obtained using the study group ‘Leipzig’ (0 control group (CG), six osteoarthritis (OA), 10 rheumatoid arthritis (RA)) as the training set for the rule generation and re-applying the respective rules to the same dataset. **(A)** Heatmap of the membership weights applying all rules of the primary rule set (α = 0.85; ‘CG’, zero rules; ‘OA’, 72 rules; ‘RA’, four rules) for the prediction of the clinical status of the different samples; dashed red lines indicate the lower limits of the respective pruned lists of rules subsequently applied in (B). **(B)** Heatmap of the membership weights applying pruned lists of rules (α = 0.85; ‘CG’, zero top-ranked rules; ‘OA’, four top-ranked rules; ‘RA’, four top-ranked rules) for optimized prediction of the clinical status of the different samples. **(C)** Confusion matrix for the rule set displayed in heatmap (B). TP, true positives; TN, true negatives; FP, false positives; FN, false negatives.

**Figure 5:**
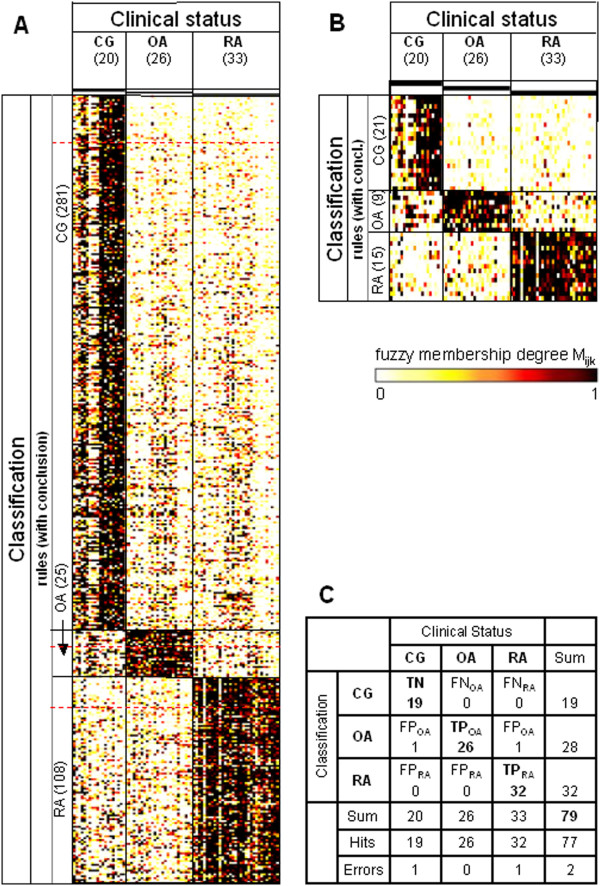
**Heatmaps and confusion matrix for the study group ‘Total’.** Data were obtained using the study group ‘Total’ (pooled data from the three centers; 20 control group (CG), 26 osteoarthritis (OA), 33 rheumatoid arthritis (RA)) as the training set for the rule generation and re-applying the respective rules to the same dataset. **(A)** Heatmap of the membership weights applying all rules of the primary rule set (α = 0.95; ‘CG’, 281 rules; ‘OA’, 25 rules; ‘RA’, 108 rules) for the prediction of the clinical status of the different samples; dashed red lines indicate the lower limits of the respective pruned lists of rules subsequently applied in **(B)**. **(B)** Heatmap of the membership weights applying pruned lists of rules (α = 0.95; ‘CG’, 21 top-ranked rules; ‘OA’, nine top-ranked rules; ‘RA’, 15 top-ranked rules) for optimized prediction of the clinical status of the different samples. **(C)** Confusion matrix for the rule set displayed in heatmap **(B)**. TP, true positives; TN, true negatives; FP, false positives; FN, false negatives.

First, each rule ‘IF *P*_*r*_ THEN *C*_*r*_’ with the premise *P*_*r*_ (*P*_*r*_ = ‘the expression *y* of gene *i* is assigned to cluster *k* (i.e., “low” or “high”)’) was weighted by application of the aforementioned fuzzy membership degree (*W*_*rj*_ = *M*_*ijk*_*′*(*y*_*ij*_)) to the sample *j* (see earlier Clustering). These membership weights (*W*_*rj*_; range from 0 to 1, with 1 indicating an unequivocal prediction of the conclusion *C*_*r*_) were visualized in a heat map for all samples (*j*) and all rules (Figures 1, 2, 3, 4, 5A,B of the respective study group).

Next, the weights *W*_*j*_(‘CG’), *W*_*j*_(‘OA’), and *W*_*j*_(‘RA’) for each individual sample *j* were calculated by summing up the respective membership weights (*W*_*rj*_) over all rules (*r*) belonging to the rule set for a given conclusion.

Finally, the highest weight was used for the prediction of the clinical status of each sample (so-called ‘defuzzification’):

$$C_{\mathrm{predict}\_j}=\arg\max\left(W_{j}\left(‘\mathrm{CG}’\right),W_{j}\left(‘\mathrm{OA}’\right),W_{j}\left(‘\mathrm{RA}’\right)\right)$$

This procedure is used for prediction of the clinical status in both the original training set (*y*_*ij*_) from a given study group (for example, ‘Jena’) and all subsequently analyzed test sets from other study groups (for example, ‘Berlin’ and ‘Leipzig’).

### Evaluation of a rule set

Comparing the predicted conclusions (C__predict_j_) with the observed clinical status (*D*_*j*_), the numbers of true positives (*TP*), true negatives (*TN*), false positives (*FP*) and false negatives (*FN*) were counted individually for the three states (‘CG’, ‘OA’, ‘RA’) to set up the confusion matrix. The sum of the *TP* and *TN* over the three states gives a number called ‘Hits’ and the sum of *FN* and *FP* a number called ‘Errors’. The total sum (*n* = *TP* + *TN* + *FP* + *FN*) equals the number of samples.

The following measures were calculated to assess the quality of the classification:

Sensitivity for the classification of RA = *TP*_*RA*_ / (*TP*_*RA*_ + *FN*_*RA*_ + *FP*_*OA*_); all values derived from the column clinical status RA in the respective confusion matrix

Sensitivity for the classification of OA = *TP*_*OA*_ / (*TP*_*OA*_ + *FN*_*OA*_ + *FP*_*RA*_); all values derived from the column clinical status OA in the respective confusion matrix

Specificity for the classification of RA = *TN*_*RA*_ / (*TN*_*RA*_ + *FP*_*RA*_); with *TN*_*RA*_ = *TN + FN*_*OA*_*+ TP*_*OA*_ + *FP*_*OA*_ (latter value derived from the column clinical status CG) and with the value for *FP*_*RA*_ representing the sum of the two corresponding fields in the columns clinical status CG and OA of the respective confusion matrix

Specificity for the classification of OA = *TN*_*OA*_ / (*TN*_*OA*_ + *FP*_*OA*_); with *TN*_*OA*_ = *TN + FN*_*RA*_*+ TP*_*RA*_*+ FP*_*RA*_ (latter value derived from the column clinical status CG) and with the value for *FP*_*OA*_ representing the sum of the two corresponding fields in the columns clinical status CG and RA of the respective confusion matrix

Overall specificity (RA + OA) = *TN/(TN + FP*_*OA*_*+ FP*_*RA*_); all values derived from the column clinical status CG in the respective confusion matrix

Accuracy = (*TN* + *TP*_*OA*_ + *TP*_*RA*_)/*n*

The sensitivities were calculated on the basis of the numbers from the corresponding columns of the confusion matrix (see above). *FN*_*RA*_ represents the number of classifications as ‘CG’ if the (‘true’) clinical state was RA, and *FN*_*OA*_ the number of classifications as ‘CG’ if the (‘true’) clinical state was OA. For the study group ‘Leipzig’, which contains no control group (‘CG’), *FP*_*RA*_ represents the misclassifications as ‘RA’, if the (‘true’) clinical status was OA, and *FP*_*OA*_ represents the misclassifications as ‘OA’, if the (‘true’) clinical status was RA.

### Identification of biologically relevant molecules

Functional relations between the genes selected by the rule-based approach (total of 57) were screened using Pathway Studio (P9, version from 18 February 2013) following identification of synonyms in GeneCard (Weizmann Institute of Science, Rehovot, Israel. In addition, gene enrichment analysis was performed using the tool DAVID [47] to identify overrepresented GO-terms or KEEG pathways for the clinical states ‘CG’, ‘OA’, or ‘RA’ in the dataset ‘Total’.

## Results

In the first step, classifiers that discriminated between ‘RA’ patients, ‘OA’ patients, and healthy controls (‘CG’) were separately trained for each of the study groups and were subsequently applied (tested) for the other study groups not initially used for training.

### Training of the classifiers

The significance level *alpha*_*S*_ were set to the default value of 0.95 for ‘Jena_all’ (*n* = 33 patients/samples) and for ‘Total’ (*n* = 79). For the other study groups, *alpha*_*S*_ was reduced to 0.94 for ‘Jena’ (*n* = 33) and ‘Berlin’ (*n* = 30) and to 0.85 for ‘Leipzig’ (*n* = 16), as described in Materials and methods. *alpha*_*S*_ thus depended on both the sample size *n* and number *m* of considered probe sets (see below).

*alpha*_*S*random_, for which at least one rule was randomly generated for each of the three conclusions, was between 0.01 and 0.10 smaller than the *alpha*_*S*_ used for generation of the primary rule sets (see Additional file 2 and Materials and methods for details).

The training results obtained for the study group ‘Jena_all’ are shown in Figure 1. After primary rule generation, 45, seven, and 27 rules were obtained for the clinical states ‘CG’, ‘OA’, and ‘RA’, respectively (that is, the numbers *r*_*max*_(‘CG’, ‘Jena_all’), *r*_*max*_(‘OA’, ‘Jena_all’), and *r*_*max*_(‘RA’, ‘Jena_all’)). The corresponding rule sets are listed in Additional file 3. For each rule (*r* = 1, …, *r*_*max*_(*C*, ‘Jena_all’)) and each sample (total of 33 patients; 10 CG, 10 OA, and 13 RA), the membership weight (*W*_r_; calculated by the fuzzy membership degree) is displayed as a heat map in Figure 1A. After pruning, seven rules were selected for each of the conclusions (Figure 1B). Figure 1C and Table 3 display the confusion matrix and quality parameters of the training results. Except for the sensitivity for OA (90%) and the accuracy (97%), all quality parameters reached 100%.

**Table 3:** **Optimized number of pruned rules (*r***_***opt***_**( *C *, *S*))**^**a **^**and assessment of training results**

**Study group *S***	**‘Jena_all’**	**‘Jena’**	**‘Berlin’**	**‘Leipzig’**	**‘Total’**
Figure	1	2	3	4	5
Number of rules for ‘CG’	7	9	8	0	21
Number of rules for ‘OA’	7	10	4	4	9
Number of rules for ‘RA’	7	10	8	4	15
Sensitivity for RA (%)	100	100	100	100	97
Sensitivity for OA (%)	90	100	100	100	100
Specificity for RA (%)	100	100	100	100	100
Specificity for OA (%)	100	100	100	100	96.2
Overall specificity (RA + OA) (%)	100	100	100	n.a.	95
Accuracy (%)	97	100	100	100	97.5

CG, control group; n.a., not applicable; OA, osteoarthritis; RA, rheumatoid arthritis. ^a^See Additional file 3.

The following results are restricted to probe sets that were qualified by a ‘present call’ for all samples of the respective dataset. In the case of the dataset ‘Jena’, a number *m* of 7,768 probe sets was considered, for ‘Berlin’ 5,159 probe sets, for ‘Leipzig’ 8,539 probe sets, and for ‘Total’ 4,982 probe sets.

Using the reduced dataset for ‘Jena’, a total of 61 rules was generated (31 rules for ‘CG’, 10 rules for ‘OA’, and 20 rules for ‘RA’) as shown in Figure 2A. This primary rule set was pruned to a set of 29 rules, whose performance is displayed in Figure 2B,C. The rule set trained with the data of the study group ‘Jena’ and applied to the same dataset resulted in zero errors (Figure 2C) and an optimization of all quality parameters to 100% (Table 3).

The same type of analysis (application of ‘present calls’; rule set training) was performed for the study groups ‘Berlin’ and ‘Leipzig’ (Figures 3 and 4; summary in Table 3). Again, rule sets trained in and re-applied to the same dataset resulted in zero errors (Figures 3C and 4C). For the study group ‘Leipzig’, however, the overall specificity could not be estimated due to missing data in the control group (‘CG’). Rule set training in the pooled 79 samples from the study groups ‘Jena’, ‘Berlin’, and ‘Leipzig’ (named study group ‘Total’) resulted in the rules displayed in Figure 5 and in only two errors (77 truly classified samples; Figure 5C).

Internal validation of pruned rule sets from the three clinical centers by leave-one-out cross-validation and bootstrapping resulted in acceptable error rates (see Additional file 2).

### Testing of the classifiers

The classifiers separately trained in the study groups ‘Jena’, ‘Berlin’, and “Leipzig’ (see Figures 2, 3 and 4) were next applied to the respective other study groups not used for training (Table 4). The average accuracy was approximately 91%, ranging from 80 to 100%. The mean sensitivity for the prediction of RA was 96%, ranging from 90 to 100%; and that for the prediction of ‘OA’ was 86%, ranging from 40 to 100%.

**Table 4:** Assessment of test results

**Training set from study group**	**‘Jena’**	**‘Jena’**	**‘Berlin’**	**‘Berlin’**	**‘Leipzig’**	**‘Leipzig’**
Test set from study group	‘Berlin’	‘Leipzig’	‘Jena’	‘Leipzig’	‘Jena’	‘Berlin’
Number of rules for ‘CG’	9	9	8	8	0	0
Number of rules for ‘OA’	10	10	4	4	4	4
Number of rules for ‘RA’	10	10	8	8	4	4
Sensitivity for RA (%)	100	100	92.3	100	92.3	90
Sensitivity for OA (%)	40	100	90	83.3	100	100
Specificity for RA (%)	100	100	80	83.3	100	100
Specificity for OA (%)	100	100	91.3	100	92.3	90
Overall specificity (RA/OA) (%)	100	n.a.	60	n.a.	n.a.	n.a.
Accuracy (%)	80	100	81.8	93.8	95.6	95
Test samples	30	16	33	16	23	20
Hits for CG	10	0	6	0	0	0
Hits for OA	4	6	9	5	10	10
Hits for RA	10	10	12	10	12	9
Hits total	24	16	27	15	22	19
Errors for CG	0	0	4	0	0	0
Errors for OA	6	0	1	1	0	0
Errors for RA	0	0	1	0	1	1
Errors total	6	0	6	1	1	1

CG, control group; n.a., not applicable; OA, osteoarthritis; RA, rheumatoid arthritis.

The number of ‘Errors’ for the prediction of RA was generally extremely small; in three cases (‘Jena’ → ‘Berlin’, ‘Jena’ → ‘Leipzig’, and ‘Berlin’ → ‘Leipzig’), no errors were detected; in the remaining cases there was only one error each (1/13, 1/13, and 1/10, respectively).

For the remaining two clinical states (that is, ‘CG’ and ‘OA’) more errors were detected. In the case of ‘Jena’ → ‘Berlin’, six OA patients were misclassified as ‘CG’; whereas in the case of ‘Berlin’ → ‘Jena’, three CG samples were misclassified as ‘RA’ and one CG sample as, OA in addition to one OA patient being misclassified as ‘RA’.

### Molecular interpretation of the obtained rule sets

The complete overlap of all rules (that is, premises and conclusion) resulting from the comparison of all study groups before pruning is shown in Additional files 3 and 4 (please note the cross-table listing of the overlapping genes in Table B of the sheet ‘Rule Overlap among Data Sets’ in Additional file 3).

If, for the purpose of identifying biologically relevant classifiers, the overlap analysis is focused on the three independent study groups ‘Jena’, ‘Berlin’, and ‘Leipzig’, a list of selected potential ‘key‘ players can be extracted (Table 5).

**Table 5:** Overlap between the three independent study groups

**Gene symbol**	**Probe set name**	**Expression level**	**‘Jena’**	**‘Berlin’**	**‘Leipzig’**	**‘Total’**
			**rule rank**	**rule rank**	**rule rank**	**rule rank**
‘CG’						
**NFIL3**	**203574_at**	**High**	**3**	**4**		1
JUND	203752_s_at	High	11	85		18
**MAT2A**	**200768_s_at**	**High**	**2**	83		5
**TIPARP**	**212665_at**	**High**	12	**8**		
LEPROTL1	202594_at	Low	27	127		113
‘RA’						
**STAT1**	**M97935_3_at [200887_s_at]**	High	19	**1** (& 10)		2 (& 10)
**GBP1**	**202270_at [202269_x_at]**	**High**	**2**	**2 (& 8)**		5 (& 6)
PSMB9	204279_at	High	13	17		1
**PLCG2**	**204613_at**	**High**	14	**5**		4
LY75	205668_at	High	12	26		8
**CSF2RB**	**205159_at**	**High**	17	28	**1**	3
**STK10**	**40420_at**	**High**	**5**	21		12

The genes belonging to at least one of the pruned rule sets of the three independent study groups are highlighted in bold, genes/rules detected by two different probe sets are indicated by numbers in parentheses.

Whereas no overlap between these groups was found for rules with the conclusion ‘OA’, remarkable overlap was found for the conclusions ‘CG’ and ‘RA’.

The rule ‘IF NFIL3 is highly expressed THEN CG’ (with NFIL3 coding for the nuclear factor interleukin-3-regulated protein) was generated with high relevance from both the ‘Jena’ and the ‘Berlin’ datasets (ranked in third and fourth position, respectively; Table 5). In addition, the two genes MAT2A (methionine adenosyltransferase 2A) and TIPARP (2,3,7,8-tetrachlorodibenvzo-*p*-dioxin (TCDD)-inducible poly(ADP-ribose) polymerase) were identified in prominent rules for ‘CG’, each only present in the pruned rule set of one study group.

For the conclusion ‘RA’, the rules concerning the ‘high’ expression of the genes STAT1, GBP1, PLCG2, CSF2RB, and STK10 were highly ranked in pruned rule sets from different study groups. STAT1 (signal transducer and activator of transcription 1) was found in the pruned rule set ‘Berlin’ (rank 1), and GBP1 (interferon-inducible guanylate binding protein 1) in the pruned rule sets ‘Jena’ (rank 2) and ‘Berlin’ (ranks 2 and 8). PLCG2 (phospholipase c-gamma-2) was found in the pruned rule set ‘Berlin’ (rank 5), and STK10 (serine/threonine kinase 10) in the pruned rule set ‘Jena’ (rank 5).

Strikingly, the relevance of the rule ‘IF CSF2RB is highly expressed THEN RA’ (CSF2RB coding for the interleukin 3 receptor/granulocyte-macrophage colony stimulating factor 3 receptor, beta was supported by three different features: the rule was independently detected in the rule sets derived from all three centers (‘Jena’, ‘Berlin’, and ‘Leipzig’); the rule occupied the highest rank (rank 1) in the rule set from ‘Leipzig’; and its complementary rule ‘IF *CSF2RB* is low THEN OA’ was also detected in the rule set ‘Leipzig’ with rank 3 (see Additional file 3).

To address a potential pathogenetic role of the genes indicated in Table 5, their expression was compared among the three different clinical states (both individually for the three different clinical centers and for the pooled study group ‘Total’ derived from all centers). In support of their relevance, all genes/rules characterizing ‘CG’ were significantly overexpressed in CG as compared with both RA and OA (Additional file 5) – with the exception of the gene/rule LEPROTL1 (leptin receptor overlapping transcript 1), which also showed significant differences, but with an opposite orientation (all *P* ≤ 0.05; Mann Whitney U test).

Strikingly, all genes/rules identified for RA also appeared highly discriminative, as shown by a significant overexpression in RA in comparison with both CG and OA (*P* values between 10^−11^ and 0.05 for 41/42 comparisons; *P* = 0.056 for the remaining comparison; Additional file 5).

In addition to the analysis of the overlapping rules, all 57 rules generated from the different study groups after pruning – that is, 29 rules trained from the dataset ‘Jena’, 20 from ‘Berlin’, and eight from ‘Leipzig’ (highlighted in the complete rule set in Additional file 3) – were screened for functional relations using Pathway Studio following identification of synonyms in GeneCard.

Since for three Affymetrix probe sets no gene names were identified (see Additional file 6), only 54 genes were analyzed using Pathway Studio. The results of the Pathway Studio search for the conclusions ‘CG’ and ‘RA’ are shown in Additional files 7 and 8, respectively.

Again, no relations were found for the conclusion ‘OA’. For ‘RA’, instead, three relations were found (Table 6). In addition to the well-known relation JAK2 → STAT1, which regards various cell types including fibroblasts (total of 70 references named by Pathway Studio), the relation STAT1 → GBP1 [48-50] and the relation JAK2 → CSF2RB [51-53] have only been addressed by a limited number of publications.

**Table 6:** Interactions between the premises/genes of the pruned rule sets generated from the ‘Jena’, ‘Berlin’, and ‘Leipzig’ data sets (total of 57 rules), as found by Pathway Studio and exemplified for the conclusion ‘RA’

**Relation**	**Type**	**Cell type**	**Number of references**
JAK2 → STAT1	Promoter binding	Various	(70)
JAK2 → CSF2RB	Regulation	Hematopoietic cells	(3)
STAT1 → GBP1	Protein modification	Fibroblasts	(3)

For more details, see Additional file 8. Pathway Studio from Elsevier, Munich, Germany.

Please note that JAK2 is not contained in Table 5 since it was only detected in the rule set for ‘RA’ in the study group ‘Jena’ (rank 3).

Gene enrichment analysis for molecular interpretation of the obtained rule sets resulted in additional information. In CG, for example, there was low expression of genes involved in MHC class II antigen processing/presentation (Additional file 9, sheets ‘CG Low BP’ and ‘CG Low KEGG’). In RA, in contrast, there was high expression of genes involved in immune response in general and leukocyte/T-cell/B-cell activation (Additional file 10, sheets ‘RA High BP’ and ‘RA High KEGG’), as well as programmed cell death (Additional file 10, sheets ‘RA High BP’, ‘RA High KEGG’, and ‘RA Low BP’).

As already observed for the sensitivity and accuracy, as well as the rule overlap and molecular interpretation, OA patients were again more difficult to discriminate, as indicated by the almost complete absence of indicative GO terms or KEGG pathways in gene enrichment analysis (Additional file 11).

## Discussion

In the present study, three multicenter, genome-wide transcriptomic datasets from a total of 79 individuals were used to infer rule-based classifiers to discriminate RA, OA, and healthy controls. In all cases, the rule sets were inferred separately from one of three centers and applied to the other centers for validation. This novel approach resulted in a high performance (close to 90% for specificity, sensitivity, and accuracy) for the discrimination of RA. Unbiased analysis of the biological relevance of the underlying rules by Pathway Studio resulted in the identification of pathways with known pathogenetic or therapeutic relevance in RA. In addition, serine/threonine kinase 10 (lymphocyte-oriented kinase) was identified as a novel molecule with a potential role in RA. Yet another novel contribution of the present study is the identification of molecules that identify normal synovial tissue, an aspect barely addressed to date.

### New approach for the identification of discriminating genes and/or rules

A novel rule-based approach was used to identify genes (in combination with their expression status) suitable for the discrimination of the clinical states healthy controls (‘CG’), ‘OA’, and ‘RA’. This approach has the major advantage of skipping the identification of differentially expressed genes on the basis of fold changes and/or *t*-test or *U*-test analysis, a process highly sensitive to heterogeneity in the patient data and therefore often incapable of identifying relevant disease-specific genes.

The rule-based approach applied in the present study is based on the relevance index of Krone and Kiendl [40]; this relevance index has so far only been used for rule generation in electrical control engineering [41] or biotechnology [38]. In addition, there are only few examples for the application of this relevance index to omics data (for example [54]) and, to our knowledge, none for the application to data in the rheumatology field.

Rule set pruning, applied in order to minimize the numbers of both rules and ‘Errors’, was successfully used to avoid overfitting and informative imbalance [55]. From our experience with heuristic rules, at least four rules per conclusion were required [38,55].

### Quality parameters of the training results

For the datasets ‘Jena’, ‘Berlin’, and ‘Leipzig’, the values for disease-oriented sensitivity and specificity, overall specificity, and accuracy were all 100%. This high performance for the training of the classifiers was expected, but still shows that this approach is suitable for the analysis of gene expression data from synovial tissue.

Interestingly, the disease-specific sensitivity for OA in the ‘Jena_all’ dataset was only 90%, resulting in an accuracy of 97% (see Table 3), whereas the quality parameters in the ‘Jena’ dataset all reached 100%. This is probably due to the highly stringent approach of only using probe sets with a ‘present call’ in all samples, deliberately chosen to minimize false positives. This approach is further supported by reduced error rates in the internal validation of the “Jena’ dataset in comparison with the ‘Jena_all’ dataset (see Additional file 2).

The results for the quality parameters in the largest possible dataset ‘Total’, containing 19 CG, 26 OA, and 32 RA, also proved highly satisfactory; that is, ≥95%. This further underlines the suitability of the relevance index approach for large-scale clinical studies with high numbers of RA and OA patients [27,30].

### Quality parameters of the test results

The quality parameters of the test results for the prediction of RA were also highly satisfactory; that is, they showed a mean close to or higher than 90% for all assessment parameters (see Table 4). This shows that the real challenge of the present study – that is, the prediction of RA in test datasets independent of the training dataset – can be met with a high accuracy and may indeed contribute to the identification of biomarkers for RA.

Notably, the mean sensitivity and specificity for the prediction for OA was considerably lower than for RA, due to both misclassification of OA as ‘CG’ (six cases) or as ‘RA’ (two cases). This is consistent with the clinical problem of properly differentiating burnt-out, possibly more heterogeneous, OA with low inflammatory activity from normal controls on one hand, and active, highly inflammatory OA from RA on the other [1,2].

### Molecular interpretation of the obtained rule sets

The number of studies aimed at identifying disease-specific signatures in rheumatology with microarray-based methods is limited [30,31,35,56-60]. Also, very few datasets addressing this question are publicly available and have been repeatedly used for bioinformatic analyses. In addition, with one exception [57], these studies have not analyzed matched multicenter datasets for rheumatic diseases. Finally, studies have resulted in the identification of numerous and heterogeneous biomarker genes or pathways with only limited overlap among the results of the different studies.

In the present study, in contrast, several rules were identified in more than one rule set generated in the three clinical centers; that is, five rules for the prediction of healthy controls (CG) and seven rules for the prediction of RA (see Table 5). Notably, a total of seven of these rules were represented not only in the primary rule set of the centers, but also in one or more of the respective pruned rule sets. Strikingly, no overlapping rules were observed for ‘OA’, again underlining the problem of properly differentiating OA from either CG or RA (see above for the Quality parameters of the test results).

In addition, automated analysis of interactions by Pathway Studio between the molecules identified in the union of all optimized rule sets (total of 57 rules; derived from three clinical centers with either two or three disease states) resulted in three interactions supported by at least three references; that is, JAK2 → STAT1 (70 references), STAT1 → GBP1 (three references) and JAK2 → CSF2RB (three references; see Table 6). Please note that JAK2 was only detected once at rank 3 in the ‘Jena’ rule set (see Additional file 3) and is therefore not listed in Table 5.

### Rules for the prediction of healthy controls (CG)

The genes identified above as overexpressed in CG may represent a core set of markers of healthy tissue and reflect regulatory genes specifically involved in the downregulation/prevention of rheumatic diseases (that is, OA or RA).

### Nuclear factor interleukin-3-regulated protein

NFIL3 is a basic leucine transcription factor acting as a regulator of genes associated with acquired and innate immunity (for example, interleukin (IL)-3 and interferon-gamma (IFNγ) [61]) or with the inhibition of proliferation and senescence [62]. In particular, NFIL-3 negatively regulates IL-12 p40 in macrophages and dendritic cells [63,64] and suppresses TH2 cytokines [65], as well as the development and maturation of NK cells [66]. In addition, NFIL3 exhibits anti-apoptotic features [67]. In particular, the role of NFIL3 in limiting the production of proinflammatory IL-12 may explain its upregulation in the normal CG. On the other hand, its prominent influence on essential cellular features (for example, metabolism, growth, viability) points to a potential contribution to the pathogenesis of RA (and/or OA) in the case of dysregulated underexpression.

### Jun D proto-oncogene

Members of the JUN and FOS families are known as immediate-early response proto-oncogenes, since they are rapidly induced by various activating agents and, on the other hand, have a very short half-life (in the range of minutes for both mRNA and protein) [68]. As in the case of NFIL3, the transcription factor JunD also regulates genes involved in acquired and innate immunity [69], in proliferation and senescence [70], or in anti-apoptotic effects [71,72].

Individual JUN/FOS family molecules show different biological activities. Whereas C-JUN and C-FOS are known as activating proto-oncogenes with transforming activity [73,74], JUND also shows de-activating features [68,73,75-77]. The effects of AP-1 complexes composed of different JUN/FOS family members clearly depend on the local promoter context of genes driven by AP-1 (for example, MMP-1 [78,79]). JUND suppresses synovial fibroblast proliferation and even antagonizes Ras-mediated transformation of the fibroblasts [77], and thus its overexpression may exert a protective role in the synovial membrane of normal joints.

### Methionine adenosyltransferase 2A

The importance of the overexpression of MAT2A in CG samples is presently unclear. This molecule is involved in the regulation of basic cellular functions, such as the synthesis of polyamines (thought to play a role in nucleic acid and protein synthesis) and developmental processes [80].

### 2,3,7,8-tetrachlorodibenzo-*p*-dioxin-inducible poly(ADP-ribose) polymerase (TIPARP)

Poly(ADP-ribosyl)ation physiologically contributes to the survival of damaged proliferating cells by immediate, DNA damage-dependent post-translational modification of histones and other proteins in the nucleus. By this process, poly(ADP-ribose) polymerases are involved in cellular functions such as proliferation and cell death. It is to be expected that the growing poly(ADP-ribose) polymerase superfamily may become the target of pharmacological strategies enhancing both antitumor efficacy and the treatment of a number of inflammatory and neurodegenerative disorders [81].

TiPARP (PARP-7) was originally identified by differential display as a TCDD-induced mRNA [82]. Although the exact function of TiPARP is presently unclear, its effects on T-cell function and its possible contribution to tumor promotion suggest a role also in the normal or arthritic synovial membrane [81].

### Leptin receptor overlapping transcript-like 1

The leptin receptor overlapping transcript (also called OB-RGRP [83]) is one of the three members of a gene family [84,85]. Leptin receptor overlapping transcript molecules are small proteins of 131 to 140 amino acids, carrying four potential transmembrane domains.

LEPROTL1, a gene widely expressed in human tissues, including metabolic tissues such as muscle and liver [83,84,86], has an influence on growth, plasma insulin-like growth factor-1 levels, hepatic sensitivity to growth hormone, and cell-surface growth hormone or leptin receptor expression and leptin signaling [87,88].

The high importance of LEPROTL1in protein trafficking to the vacuole/lysosome of eukaryotic cells, a process initially regarded as pathogenetically relevant in RA [89-91], and in the downregulation of membrane protein levels suggests a phylogenetically conserved role for LEPROTL1 [85]. Since LEPROTL1 does not appear to act as a classic leptin receptor, its role in the physiology and pathophysiology of the synovial membrane is presently uncertain.

In the present dataset, the above-mentioned NFIL3, JUND, MAT2A, and TIPARP were indeed significantly overexpressed in the synovial membrane of CG as compared with both RA and OA (both individually for the three different clinical centers and for the pooled study group ‘Total’ derived from all centers; Additional file 5). Interestingly, overexpression of JUND (OA vs. RA) has not only been observed in synovial membranes, but also in proinflammatory synovial fibroblasts isolated from synovial tissue [92].

In contrast, LEPROTL1 was the only gene significantly underexpressed in the synovial membrane of CG as compared with both RA and OA, suggesting that this molecule may support inflammatory and/or degenerative joint diseases. Similarly to JUND, however, in an opposite direction, differential expression of LEPROTL1 was not only observed in synovial membranes, but also in resident synovial fibroblasts [92].

### Rules for the prediction of rheumatoid arthritis

The genes overexpressed in RA synovial tissue (see Table 5) may represent biomarkers of RA and reflect processes specifically involved in the pathogenesis and/or progression of the disease. A disease specificity of the markers is strongly supported by their significant overexpression in RA, not only in comparison with CG but also with the ‘disease’ control OA (see Additional file 5). In the RA groups, genes especially associated with the regulation of immunologic processes appear to be suitable as disease-specific identifiers.

### Signal transducer and activator of transcription 1

STAT1, a transcription factor regulating (amongst others) immunity-mediating genes, is known to be upregulated in RA patients [59,93]. In addition to other transcription factors (for example, NFKB or AP-1), STAT1 has long been regarded as a pivotal transcription factor involved in joint inflammation and destruction [60,94]. The identification of these key factors underlines the robustness of the present approach. This is further underlined by the fact that the rule ‘STAT1 high in RA’ appears a total of five times in three different rule sets (rule set ‘Jena’, position 19; rule set ‘Berlin’, positions 1 and 10; rule set ‘Total’, positions 2 and 10; see Table 5 and Additional file 4 for details and the corresponding Affymetrix probe sets).

In addition, there was a reciprocal detection of the complementary rule ‘IF STAT1 is low THEN OA’ in the rule set ‘Leipzig’ with rank 12 (see Additional file 3).

### Interferon-inducible guanylate binding protein 1

GBP1, a protein specifically binding guanylated nucleotides with potential effects on GTPases involved in signal transduction, has been already described as upregulated in RA versus OA synovial tissue [95]. Also, this factor is implicated in the pathogenesis of RA due to its upregulation by IFNγ [95,96]. As in the case of STAT1, this finding confirms that key mediators of rheumatoid inflammation have been identified in the present study. This is again further underlined by the fact that the rule ‘GBP1 high in RA’ appears a total of five times in three different rule sets (rule set ‘Jena’, position 2; rule set ‘Berlin’, positions 2 and 8; rule set ‘Total’, positions 5 and 6; see Table 5 and Additional file 4).

### Proteasome (prosome, macropain) subunit, beta type, 9 (large multifunctional peptidase 2/low molecular mass protein 2)

The proteasomal subunit PSMB9 (also known as LMP2; see abbreviations) is involved in the regulation of proteolytic specificity, especially in response to IFN-γ, thus enabling the formation of immunoproteasomes and the generation of peptides presentable by MHC I molecules [97]. PSMB9 also enhances cytokine production (for example, tumor necrosis factor, IL-1β, IFNγ [98]). Indeed, this molecule shows a significant genetic association with RA in ethnic Han Chinese from Yunan [99] and is the target of autoimmune reactions in RA [100]. As for STAT1 and GBP1, the validity of the rule ‘PSMB9 high in RA’ is emphasized by the fact that it appears in three different rule sets (rule set ‘Berlin’, position 13; rule set ‘Leipzig’, position 17; rule set ‘Total’, position 1; see Table 5 and Additional file 4).

### Phospholipase C-gamma-2

The function of members of the phospholipase C family is the hydrolysis of phospholipids into fatty acids and other lipophilic molecules. The family members are grouped into several subtypes and catalyze the hydrolysis of phosphatidylinositol 4,5-bisphosphate to inositol 1,4,5-trisphosphate and 1,2-diacylglycerol, which both have important second messenger functions. Phospholipase C-gamma is activated by phosphorylation in response to various growth factors or immune signals, is broadly expressed, and carries diverse biological functions in inflammation, cell growth, signaling/death, and maintenance of membrane phospholipids. Activating mutations in the PLCG2 gene have been shown to induce autoimmunity, inflammation, and/or inflammatory arthritis in murine models [101,102]. PLCG2 has already been recognized as an excellent discriminator of RA against other types of arthritis or autoimmune diseases [103] and appears to be significantly upregulated in RA synovial tissue as compared with the normal synovial membrane [104]. As for STAT1, GBP1, and PSMB9/LMP2, the validity of the rule ‘PLCG2 high in RA’ was emphasized by its appearance in three independently established rule sets (rule set ‘Berlin’, position 5; rule set ‘Jena’, position 14; rule set ‘Total’, position 4; see Table 5 and Additional file 4).

### Lymphocyte antigen 75

Ly75, a member of the human macrophage mannose receptor family (also known as DEC205 or GP200-MR6), supports antigen presentation of dendritic cells [105] and mediates anti-proliferative as well as promaturational effects in B lymphocytes [106]. This molecule is apparently upregulated in monocytes derived from RA patients in comparison with those from normal donors [107], but its role in disease is currently unknown. Interestingly, however, single nucleotide polymorphisms of the Ly75 antigen belong to the three single nucleotide polymorphisms most significantly associated with type 2 diabetes mellitus, leaving open a possible role of Ly75 in inflammatory disease [108].

### CSF2RB (interleukin 3 receptor/granulocyte macrophage colony stimulating factor 3 receptor, beta)

A most striking finding in the present study is the rule ‘CSF2RB high in RA’. CSF2RB codes for a transmembrane protein and acts as a common receptor subunit (also known as common beta chain) for granulocyte–macrophage colony-stimulating factor (GM-CSF), IL-5, and IL-3, which play a preeminent role in inflammation and hematopoiesis [109,110]. One of the ligands of CSF2RB (that is, GM-CSF) has long been implicated in the pathogenesis of RA, and other rheumatic or autoimmune diseases [60,111-119]. This has recently led to the development of neutralizing therapeutic monoclonal antibodies specifically directed against the α-chain of the GM-CSF receptor, which have been successfully used for the treatment of RA [120-122].

Notably, the rule ‘CSF2RB high in RA’ appeared in the independently established rule sets of all analyzed cohorts (rule set ‘Jena’, position 17; rule set ‘Berlin’, position 28; rule set ‘Leipzig’, position 1; and, remarkably, rule set ‘Total’, position 3), again underling the validity of the completely unbiased procedure of rule set generation. As in the case of STAT1, there was a reciprocal detection of the complementary rule ‘IF CSF2RB is low THEN OA’ in the rule set ‘Leipzig’ with rank 3 (Additional file 3).

### Serine/threonine kinase 10 (lymphocyte-oriented kinase)

STK10 is a member of the Ste20 family of serine/threonine protein kinases with similarity to several known polo-like kinase kinases [123], which associates with and phosphorylates polo-like kinase 1 and whose functional inactivation interferes with normal cell cycle progression. STK10 also negatively regulates IL-2 expression in T cells via the mitogen-activated protein kinase kinase 1 pathway [124]. Interestingly (and potentially relevant for RA), STK10 is involved in the regulation of cytoskeletal rearrangement through phosphorylation of the ezrin–radixin–moesin proteins [125], a process also strongly emphasized by a previous report [96] and by a relatively low expression of the respective genes in the gene enrichment analysis in the ‘CG’ group (see Additional file 9; sheet ‘CG low BP’). In addition, STK10 potentiates dexamethasone-induced apoptosis [126] and may thus contribute to the dysregulation of apoptosis possibly involved in RA [127]. Finally, STK10 may play a role in autoimmune skin diseases [128], although a direct involvement of this molecule in arthritis has never been reported.

As in the case of rules for healthy control (CG), all genes used for the prediction of RA were indeed significantly overexpressed in the synovial membrane of RA as compared with both OA and CG (both individually for the three different clinical centers and for the pooled study group ‘Total’ derived from all centers; see Additional file 5). Interestingly, highly significant overexpression of CSF2RB (RA vs. OA; *P* = 5.4 × 10^−6^) was not only observed in synovial membranes, but also in proinflammatory synovial fibroblasts isolated from synovial tissue [92].

Finally, in combination with JAK2, one of the most influential rules in the ‘Jena’ RA group (position 3; high in RA), a subset of the genes (STAT1, GBP1, CSF2RB) can be combined in a JAK/STAT-dependent gene regulatory network [59,60,129-131]. This also indicates that the rules identifying RA patients in the present study are not generated randomly, but reflect a mechanistic relevance within the context of RA pathogenesis. Concerning JAK2, its concrete relevance in RA is stressed by the development of therapeutic approaches directed at the JAK pathway [129].

Overall, the present study confirmed the involvement of partially or well-known molecules/pathways in RA (for example, STAT1, GBP1, PLCG2, CSF2RB), but also identified molecules previously not associated with RA (for example, STK10). Also, to our knowledge, there are at present no reports on molecules/pathways positively identifying the clinical status ‘CG’ in general, and the NFIL-3 pathway in particular. Finally, the present study presents for the first time a ‘unifying hypothesis’ by addressing the overlap of the highly ranked rules/genes among different clinical centers and thus pinning down molecules of universal relevance in heterogeneous patient cohorts from different centers. This is also supported by the representation of the top 12 rules of the ‘Total’ dataset in the overlap table; that is, the largest independently analyzed cohort in the present study (total of 79 donors (patients).

## Conclusions

In this study, three multicenter, genome-wide transcriptomic datasets were applied to infer rule-based classifiers/genes to discriminate RA, OA, and healthy controls, and were subsequently analyzed for their biological relevance using Pathway Studio and gene enrichment analysis. This novel approach resulted in a high performance for the discrimination of RA and the identification of factors with known pathogenetic or therapeutic relevance in RA (for example, STAT1, GBP1, IFNγ, GM-CSF, and its receptor CSF2RB, as well as JAK2, the latter pointing to a JAK/STAT-dependent gene regulatory network). This indicates that the rules identifying RA patients were not generated randomly, but reflect (disease-specific) key biomarkers with mechanistic relevance for RA pathogenesis and progression, some of them well established and already exploited for therapeutic purposes.

The present study contributes to focusing the diagnostic and therapeutic interest in RA on relevant and innovative molecules or pathways; for example, GM-CSF and its receptor CSF2RB. The fact that such known pathways have been identified in the present study for the prediction of RA suggests a high sensitivity and validity of the current approach. In addition, the present study for the first time addressed a multicenter cross-validation and may thus contribute to the identification of molecules with universal relevance in heterogeneous RA patient cohorts, possibly including the previously undescribed STK10.

At a molecular level, the biomarkers were significantly overexpressed in RA synovial tissue (mostly in the study groups from all three centers), not only in comparison with healthy controls, but also with the ‘disease’ control OA. In addition, significant overexpression was not limited to the synovial tissue as a whole, but also occurred in isolated synovial fibroblasts, a cell population regarded as highly important for chronic inflammatory RA.

In perspective, validation, refinement, and generalization of the present rule-based, discriminative procedure in a larger prospective cohort are necessary. The identified biomarkers may prove useful for diagnosis or differential diagnosis of RA patients (including potential subpopulations), as well as for stratification and monitoring of (responders and nonresponder) patients in routine or experimental clinical trials.

## Abbreviations

CG: control group; Cr: conclusion of the *r*th rule; CSF2RB: interleukin 3 receptor/granulocyte-macrophage colony stimulating factor 3 receptor, beta; GBP1: interferon-inducible guanylate binding protein 1; GM-CSF: granulocyte–macrophage colony-stimulating factor; IFNγ: interferon-gamma; IL: interleukin; JUND: jun D proto-oncogene; LEPROTL1: leptin receptor overlapping transcript-like 1; LY75: lymphocyte antigen 75; MAT2A: methionine adenosyltransferase 2A; NFIL3: nuclear factor interleukin-3-regulated protein; OA: osteoarthritis; PLCG2: phospholipase C-gamma-2; Pr: premise of the *r*th rule; PSMB9/LMP2: proteasome (prosome, macropain) subunit, beta type, 9 (large multifunctional peptidase 2)/low molecular mass protein 2; RA: rheumatoid arthritis; RI: relevance index; STAT1: signal transducer and activator of transcription 1; STK10: serine/threonine kinase 10 (lymphocyte-oriented kinase); TCDD: 2,3,7,8-tetrachlorodibenzo-*p*-dioxin.

## Competing interests

The authors declare that they have no competing interests.

## Author’s contributions

DW, PK, MP, RG, and DD performed the bioinformatic analysis, contributed to the design of the study, and participated in the writing and finalization of the manuscript. RWK, RG, PS, RH, and TH contributed to the design of the study and participated in the layout, writing, and finalization of the manuscript. RH, DP, TH, PS, DK, and RWK designed or performed the experiments and participated in writing and finalization of the manuscript. All authors read and approved the final manuscript.

## Supplementary Material

Additional file 1 Calculation of the relevance index. Click here for file

Additional file 2 Internal validation of rule sets. Click here for file

Additional file 3 **List of the ‘complete primary rule sets’ for all datasets, as well as the ‘Rule overlap among data sets’.** The data are displayed as either ‘complete primary rule sets’ with the pruned (optimized) rules highlighted in bold (Sheet 1) or as the ‘Rule Overlap among Data Sets’ (Sheet 2) with the rules/genes showing an overlap between the three independent study groups ‘Jena’, ‘Berlin’, and ‘Leipzig’ highlighted in grey. In both cases, the rules were generated as stated in Materials and methods (‘Rule set generation’) and the ranks of the individual rules in the respective dataset are indicated. Click here for file

Additional file 4 **Listing of the overlap among the different rule sets.** The data are displayed as the ‘Rule Overlap among Data Sets’ including the gene names. The ranks of the individuals rules in the respective dataset are indicated and the rules/genes showing an overlap between the three independent study groups ‘Jena’, ‘Berlin’, and ‘Leipzig’ are highlighted in grey. Click here for file

Additional file 5 **Log-fold change and *P* values for differentially expressed genes.** Log-fold change (log2 FC) and *P* values (Mann Whitney U test, red: *P* ≥ 0.05) for the genes differentially expressed among patients with a different clinical status (genes significantly overexpressed in RA versus both CG and OA are highlighted in grey; see also Table 6). Click here for file

Additional file 6 Genes (original symbols) and the synonyms used as input for the Pathway Studio 9 search for interactions among the genes. Click here for file

Additional file 7 **Interactions among the genes in the pruned rule sets (CG).** Interactions found by Pathway Studio among the genes contained in the pruned rule sets of the ‘Jena’ and ‘Berlin’ datasets for the conclusion ‘CG’. Click here for file

Additional file 8 **Interactions among the genes in the pruned rule sets (RA).** Interactions found by Pathway Studio among the genes contained in the pruned rule sets of the ‘Jena’, ‘Berlin’ and ‘Leipzig’ datasets for the conclusion ‘RA’. Click here for file

Additional file 9 **Gene enrichment analysis for molecular interpretation (CG).** Gene enrichment analysis for molecular interpretation of the obtained rule set for the conclusion ‘CG’ using the GO terms biological process (BP) and molecular function (MF), as well as KEGG pathways. The analyses were performed separately for the ‘CG’ rules showing a high or low expression level. Category = type of term (GO term/KEEG pathway); Term = denomination of term (interesting terms highlighted in grey); Count = list hits; number of genes in the rule set belonging to the term in question; p value = EASE score (upper boundary of the distribution of Jackknife Fisher exact probabilities given the actual Count, List Total, Pop Hits, and Pop Total); Genes = gene symbols of included rules/genes; List Total = number of genes in the rule set (for high and low expression, respectively); Pop Hits = number of genes in the population background belonging to the specific term; Pop Total = number of genes in the population background; BH-adjusted p value = Benjamini-Hochberg adjusted *P* value (threshold *P* ≤ 0.05 indicated by fat frame). Click here for file

Additional file 10 **Gene enrichment analysis for molecular interpretation (RA).** Gene enrichment analysis for molecular interpretation of the obtained rule set for the conclusion ‘RA’ using the GO terms biological process (BP) and molecular function (MF), as well as KEGG pathways. The analyses were performed separately for the ‘RA’ rules showing a high or low expression level. In the case of ‘RA’ rules showing a low expression level, there were only results for the GO terms BP and MF. Category = type of term (GO term/KEEG pathway); Term = denomination of term (interesting terms highlighted in grey); Count = list hits; number of genes in the rule set belonging to the term in question; p value = EASE score (upper boundary of the distribution of Jackknife Fisher exact probabilities given the actual Count, List Total, Pop Hits, and Pop Total); Genes = gene symbols of included rules/genes; List Total = number of genes in the rule set (for high and low expression, respectively); Pop Hits = number of genes in the population background belonging to the specific term; Pop Total = number of genes in the population background; BH-adjusted p value = Benjamini-Hochberg adjusted *P* value (threshold *P* ≤ 0.05 indicated by fat frame). Click here for file

Additional file 11 **Gene enrichment analysis for molecular interpretation (OA).** Gene enrichment analysis for molecular interpretation of the obtained rule set for the conclusion ‘OA’ using the GO terms biological process (BP) and molecular function (MF), as well as KEGG pathways. The analyses were performed separately for the ‘OA’ rules showing a high or low expression level. There were only results for the GO term MF in ‘OA’ rules showing a high expression level. Category = type of term (GO term/KEEG pathway); Term = denomination of term; Count = list hits; number of genes in the rule set belonging to the term in question; p value = EASE score (upper boundary of the distribution of Jackknife Fisher exact probabilities given the actual Count, List Total, Pop Hits, and Pop Total); Genes = gene symbols of included rules/genes; List Total = number of genes in the rule set (for high and low expression, respectively); Pop Hits = number of genes in the population background belonging to the specific term; Pop Total = number of genes in the population background; BH-adjusted p value = Benjamini-Hochberg adjusted *P* value. Click here for file
