# Supplementary material for: Identification of rheumatoid arthritis and osteoarthritis patients by transcriptome-based rule set generation
Source: Arthritis Res Ther. 2014 Apr 1;16(2):R84. doi: 10.1186/ar4526 (PMC4060460; doi:10.1186/ar4526)
Supplement: Additional file 1 — Calculation of the relevance index. [file ar4526-S1.doc]

Additional File A1

Identification of rheumatoid arthritis and osteoarthritis patients by transcriptome-based rule set generation

Woetzel, Dirk^1^; Huber, Rene^2,3^; Kupfer, Peter^4^; Pohlers, Dirk^2,5^; Pfaff, Michael^1,6^; Driesch, Dominik^1^; Häupl, Thomas^7^; Koczan; Dirk^8^; Stiehl, Peter^9^; Guthke, Reinhard^4^; Raimund W. Kinne^2^

^1^BioControl Jena GmbH, Wildenbruchstr. 15, 07745 Jena, Germany

^2^Experimental Rheumatology Unit, Department of Orthopedics, Jena University Hospital, Waldkrankenhaus Rudolf Elle, Klosterlausnitzer Str. 81, 07607 Eisenberg, Germany

^3^Institute of Clinical Chemistry, Hannover Medical School, Carl-Neuberg-Str. 1, 30625 Hannover, Germany

^4^Leibniz Institute for Natural Product Research and Infection Biology – Hans Knöll Institute, Beutenbergstr. 11a, 07745 Jena, Germany

^5^Present address: Center of Diagnostics GmbH, Chemnitz Hospital, Chemnitz, Germany

^6^Department of Medical Engineering and Biotechnology, University of Applied Sciences Jena, Carl-Zeiss-Promenade 2, 07745 Jena, Germany

^7^Department of Rheumatology and Clinical Immunology, Charite-Universitätsmedizin Berlin, Chariteplatz 1, 10117 Berlin, Germany

^8^Institute of Immunology, University of Rostock, Schillingallee 68, 18057 Rostock, Germany

^9^Institute of Pathology, University of Leipzig, Liebigstr. 24, 04103 Leipzig, Germany

**Calculation of the relevance index *RI***

For all rules the relevance index *RI* was calculated according to Troschke [39] based on the probabilities *p_Cr_* and *p_CPr_* as well as its confidence interval borders *pCr_low, pCr_high, pCPr_low,* and *pCPr_high* as follows.

First, for the rule IF *P_r_* THEN *C_r_* the following numbers were counted and calculated by summarizing the relevant membership degrees (*M_ijk_*), respectively:

n = # all samples

a = # (*P_r_* true) AND (C*_r_* true) = *TP*

b = # (*P_r_* true) – a = *FP*

c = # (C*_r_* true) – a = *FN* b+c = Errors

d = n – a – b – c = *TN* a+d = Hits

Using the before mentioned numbers, the probability *p_Cr_* of the conclusion *C_r_* and the conditional probability *p_CPr_* of the conclusion *C_r_* under the premise *P_r_* were calculated:

$$p_{Cr} = \frac{a+c}{n}$$

$$p_{C\Pr} = \frac{a}{a+b}$$

Next, the upper bound of the confidence interval of the probability *p_Cr_* and the lower bound of the conditional probability *p_CPr_* were computed:

$$\begin{matrix} p_{Cr\_high}\left(N,M,alpha\right) = \frac{\left(M+1\right)\times F\left(N_{1},N_{2},alpha\right)}{\left(N-M+\left(M+1\right)\times F\left(N_{1},N_{2},alpha\right)\right)} \\ with \\ N = n \\ M = a+c \\ N_{1} = 2\times\left(M+1\right) \\ N_{2} = 2\times\left(n-M\right) \end{matrix}$$

$$\begin{matrix} p_{C\Pr\_low}\left(N,M,alpha\right) = 1-\frac{\left(M_{1}+1\right)\times F\left(N_{1},N_{2},alpha\right)}{\left(N-M_{1}+\left(M_{1}+1\right)\times F\left(N_{1},N_{2},alpha\right)\right)} \\ with \\ N = a + b \\ M = a \\ M_{1} = N-M \\ N_{1} = 2\times\left(M_{1}+1\right) \\ N_{2} = 2\times\left(n-M_{1}\right) \end{matrix}$$

The F-quantile was approximated according to Carter [A1] as follows:

Finally, the Relevance Index *RI* results as

$$RI_{r} =\left\{\begin{matrix} \frac{p_{C\Pr\_low}-p_{Cr\_high}}{(1-p_{Cr}) p_{C\Pr}} if p_{C\Pr}>p_{Cr} and p_{C\Pr\_low}>p_{Cr\_high} \\ \\ 0 else \end{matrix}\right.$$

# References

A1. Carter A: **Approximation to percentage points of the z-distribution**. *Biometrika* 1947*,* **34**:352-358.
