# Supplementary material for: Identification of rheumatoid arthritis and osteoarthritis patients by transcriptome-based rule set generation
Source: Arthritis Res Ther. 2014 Apr 1;16(2):R84. doi: 10.1186/ar4526 (PMC4060460; doi:10.1186/ar4526)
Supplement: Additional file 2 — Internal validation of rule sets. [file ar4526-S2.doc]

**Additional File A2**

**Identification of rheumatoid arthritis and osteoarthritis patients by transcriptome-based rule set generation**

Woetzel, Dirk^1^; Huber, Rene^2,3^; Kupfer, Peter^4^; Pohlers, Dirk^2,5^; Pfaff, Michael^1,6^; Driesch, Dominik^1^; Häupl, Thomas^7^; Koczan; Dirk^8^; Stiehl, Peter^9^; Guthke, Reinhard^4^; Raimund W. Kinne^2^

^1^BioControl Jena GmbH, Wildenbruchstr. 15, 07745 Jena, Germany

^2^Experimental Rheumatology Unit, Department of Orthopedics, Jena University Hospital, Waldkrankenhaus Rudolf Elle, Klosterlausnitzer Str. 81, 07607 Eisenberg, Germany

^3^Institute of Clinical Chemistry, Hannover Medical School, Carl-Neuberg-Str. 1, 30625 Hannover, Germany

^4^Leibniz Institute for Natural Product Research and Infection Biology – Hans Knöll Institute, Beutenbergstr. 11a, 07745 Jena, Germany

^5^Present address: Center of Diagnostics GmbH, Chemnitz Hospital, Chemnitz, Germany

^6^Department of Medical Engineering and Biotechnology, University of Applied Sciences Jena, Carl-Zeiss-Promenade 2, 07745 Jena, Germany

^7^Department of Rheumatology and Clinical Immunology, Charite-Universitätsmedizin Berlin, Chariteplatz 1, 10117 Berlin, Germany

^8^Institute of Immunology, University of Rostock, Schillingallee 68, 18057 Rostock, Germany

^9^Institute of Pathology, University of Leipzig, Liebigstr. 24, 04103 Leipzig, Germany

## Internal validation of rule sets

# 1 Aim

The pruned rule sets, i.e. generated and optimised on the data of an individual study group (‘Jena_all’, ‘Jena’, ‘Berlin’, Leipzig’, or ‘Total’) group were validated using the training data set by different methods:

1. Resampling
2. Leave-one-out Cross Validation
3. Bootstrapping

# 2 Methods

**Resampling**

The significance level alpha_Srandom_ was estimated where at least one rule with a relevance index *RI* > 0 was generated for each of the three conclusions using original pre-processed gene expression values *y_ij_*, and a random assignment to the individual conclusions (‘CG’, ’RA’, and ‘OA’) in the training set.

Then, the significance level alpha_S_ used was choosen so that alpha_S_ - alpha_Srandom_  > 0.01.

### Rule set optimization

The rule set was puned by minimizing the Model error, i.e., the number of errors devided by the number of samples.

**Leave-one-out Cross Validation**

In each study group, the data of one sample were excluded for training but applied for testing. This procedure was repeated so that each sample was used one time for testing and the remaining data for training. The generalized error (mean number of errors divided by the total number of samples of the respective study group) was calculated.

**Bootstrapping E0**

The generalized error was estimated using 250 Bootstrap data sets.

# 3 Results

The **Table A2** shows the validation results.

# Table A2

Quality parameters of the rule sets

α_S_: significance level used for the generation of the primary rule set (α_S_ > α_Srandom_); α_Srandom_: significance level where at least one rule was generated for each of the three conclusions using original pre-processed gene expression values, an alpha_Srandom_, and a random assignment to the individual conclusions (‘CG’, ’RA’, and ‘OA’) in the respective data set; Error rate: Error / n for the original data; n = number of samples; Generalized error rate obtained by the Leave-one-out Cross Validation (over the number of n samples) and Bootstrapping (E0: error rate over n = 250 bootstrap data sets; E.632: weighted sum of 0.632 x E0 + 0.368 x Error rate for the original data)

| **Study group** | **α_s_** | **α_srandom_** | **Error rate** | **Generalised Error rate** | | |
| --- | --- | --- | --- | --- | --- | --- |
|  |  |  |  | **Leave-one-out Cross Validation** | **E0**  **Bootstrap** | **.632 Bootstrap** |
| Jena_all | 0.95 | 0.91 | 0.0303 | 0.242 | 0.232 | 0.157 |
| Jena | 0.94 | 0.85 | 0 | 0.182 | 0.219 | 0.138 |
| Berlin | 0.94 | 0.84 | 0 | 0.233 | 0.081 | 0.0512 |
| Leipzig | 0.85 | 0.82 | 0 | 0.1875 | 0.284 | 0.1798 |
| Total | 0.95 | 0.84 | 0.0253 | 0.0759 | 0.097 | 0.0706 |

The **Additional** **Figures A2.1** to **A2.11** show the distribution of rules obtained by the 250 bootstrap data sets. The red coloured bins representing the rules of the respective optimised rule set demonstrate that these rules are found with high frequency and, thus, are stable.


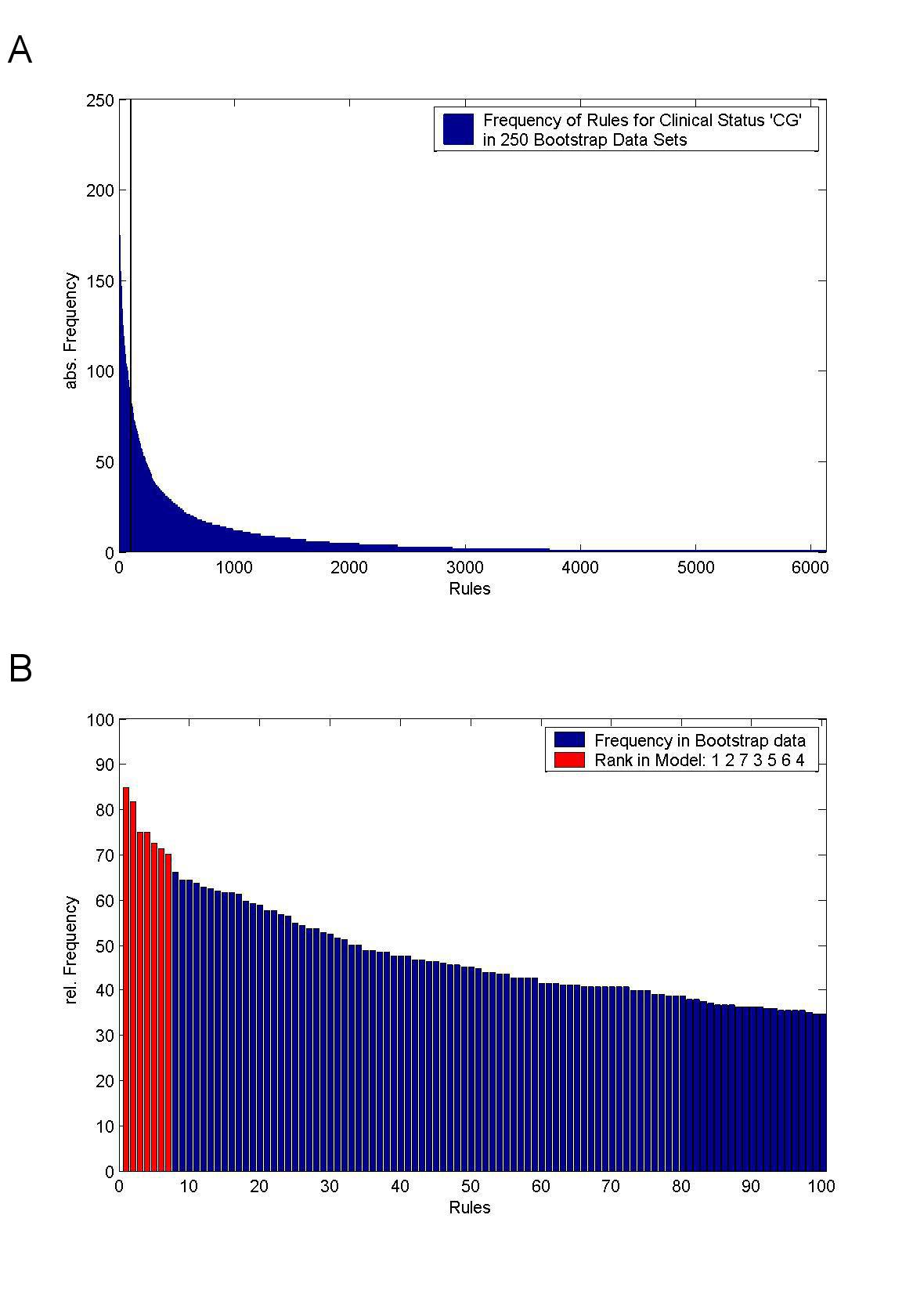


**Additional Figure A2.1**

Result of the internal validation of trained rule set for study group ‘Jena_all’ and conclusion ‘CG’. A: Absolute frequency of rules obtained from 250 Bootstrap Data Sets; B: Relative frequency of the 100 most frequent rules obtained from 250 Bootstrap Data Sets; red: the 7 rules of the optimized (pruned) rule set set (with rank 1, 2, 7, 3, 5, 6, 4).


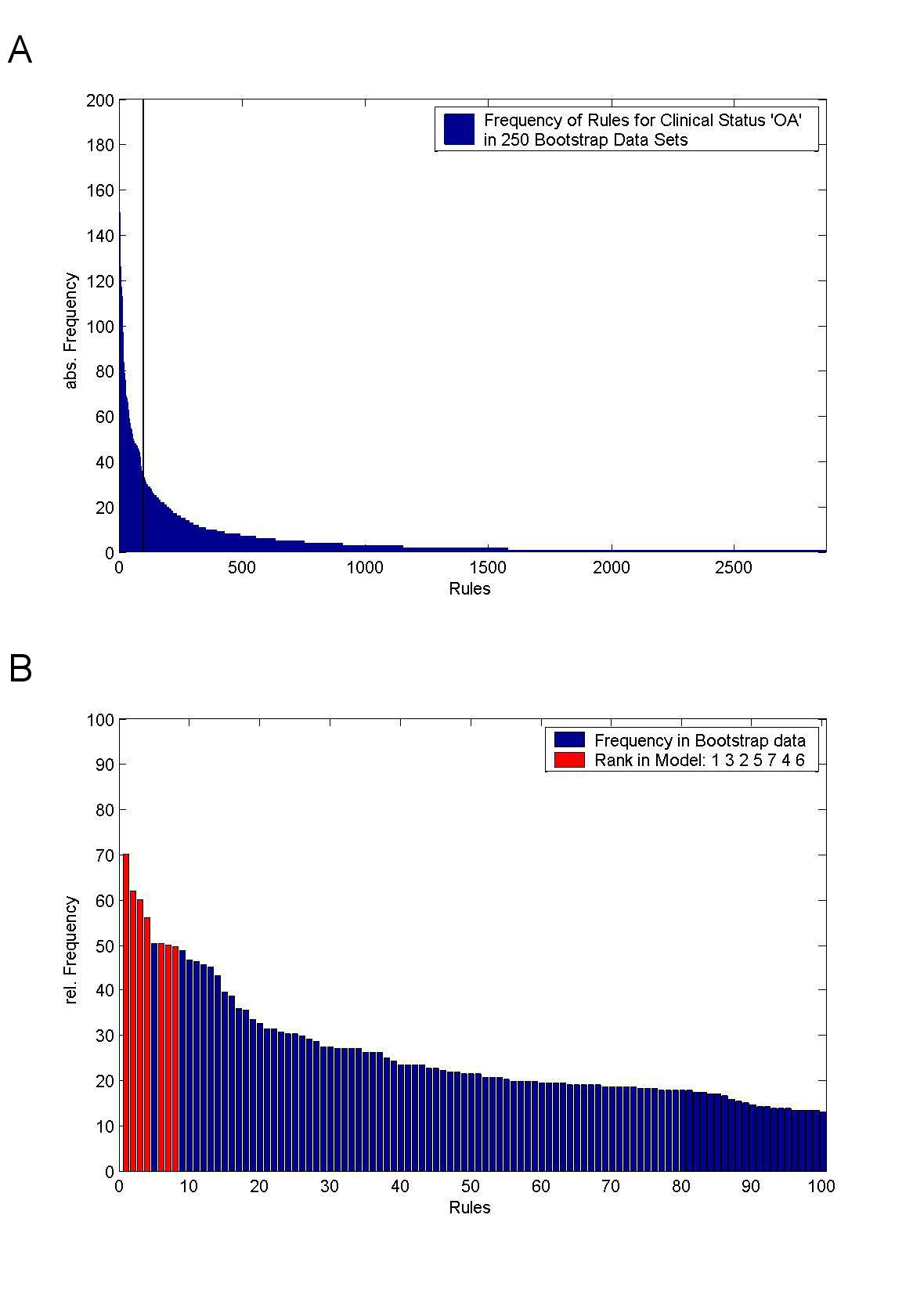


**Additional Figure A2.2**

Result of the internal validation of trained rule set for study group ‘Jena_all’ and conclusion ‘OA’. A: Absolute frequency of rules obtained from 250 Bootstrap Data Sets; B: Relative frequency of the 100 most frequent rules obtained from 250 Bootstrap Data Sets; red: the 7 rules of the optimized (pruned) rule set set (with rank 1, 3, 2, 5, 7, 4, 6).


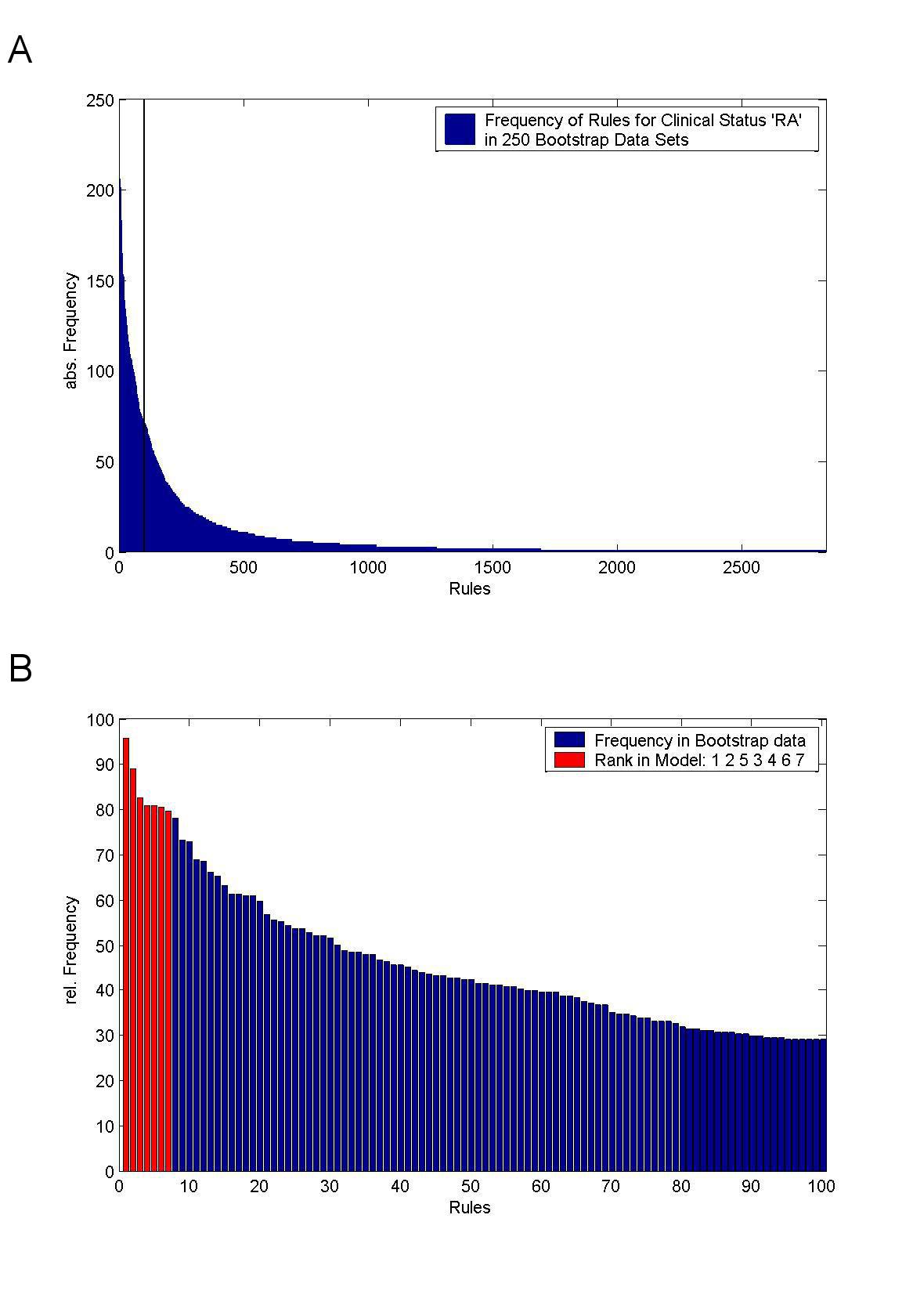


**Additional Figure A2.3**

Result of the internal validation of trained rule set for study group ‘Jena_all’ and conclusion ‘RA’. A: Absolute frequency of rules obtained from 250 Bootstrap Data Sets; B: Relative frequency of the 100 most frequent rules obtained from 250 Bootstrap Data Sets; red: the 7 rules of the optimized (pruned) rule set set (with rank 1, 2, 5, 3, 4, 6, 7).


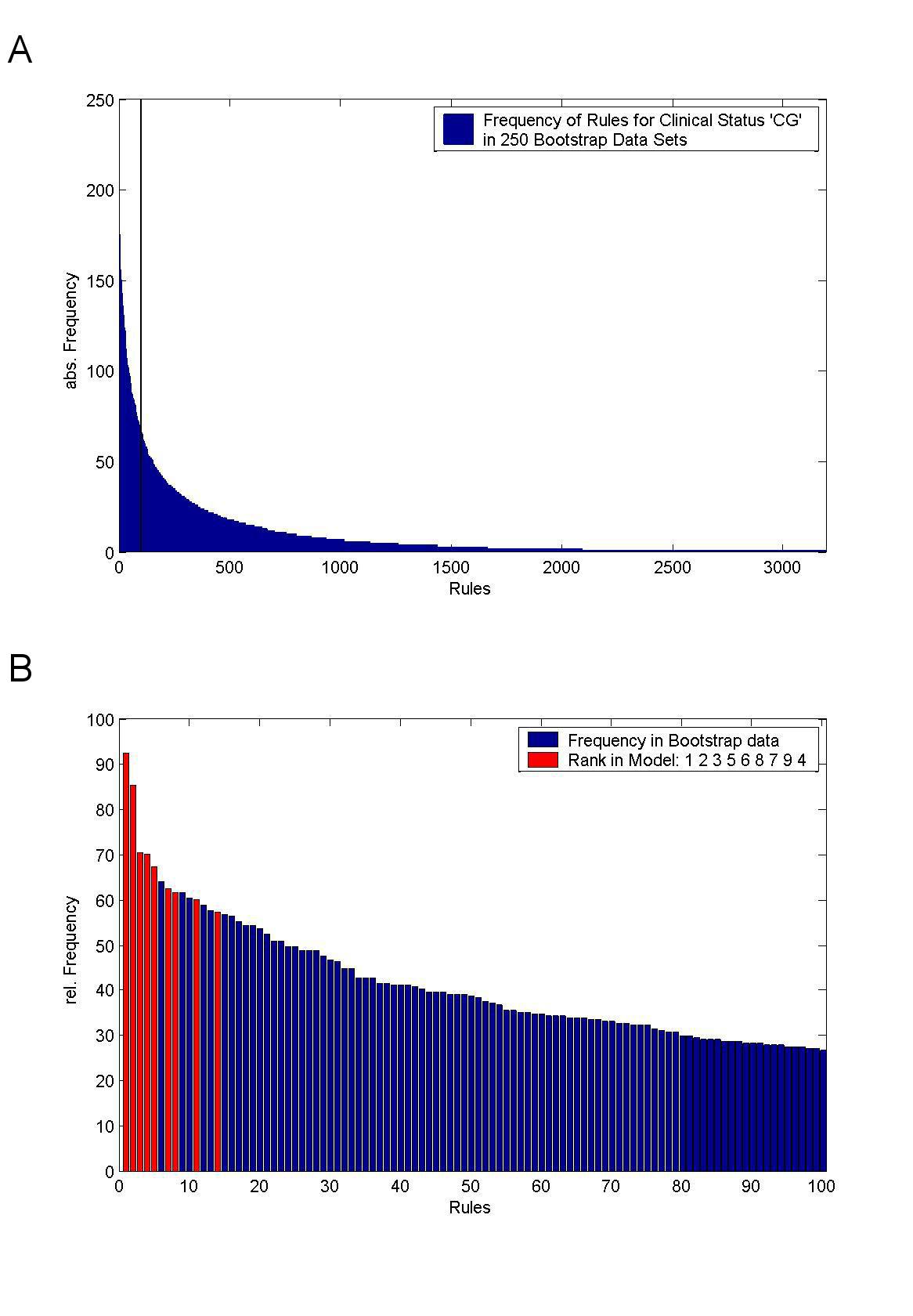


**Additional Figure A2.4**

Result of the internal validation of trained rule set for study group ‘Jena’ and conclusion ‘CG’. A: Absolute frequency of rules obtained from 250 Bootstrap Data Sets; B: Relative frequency of the 100 most frequent rules obtained from 250 Bootstrap Data Sets; red: the 9 rules of the optimized (pruned) rule set (with rank 1, 2, 3, 5, 6, 8, 7, 9, 4).


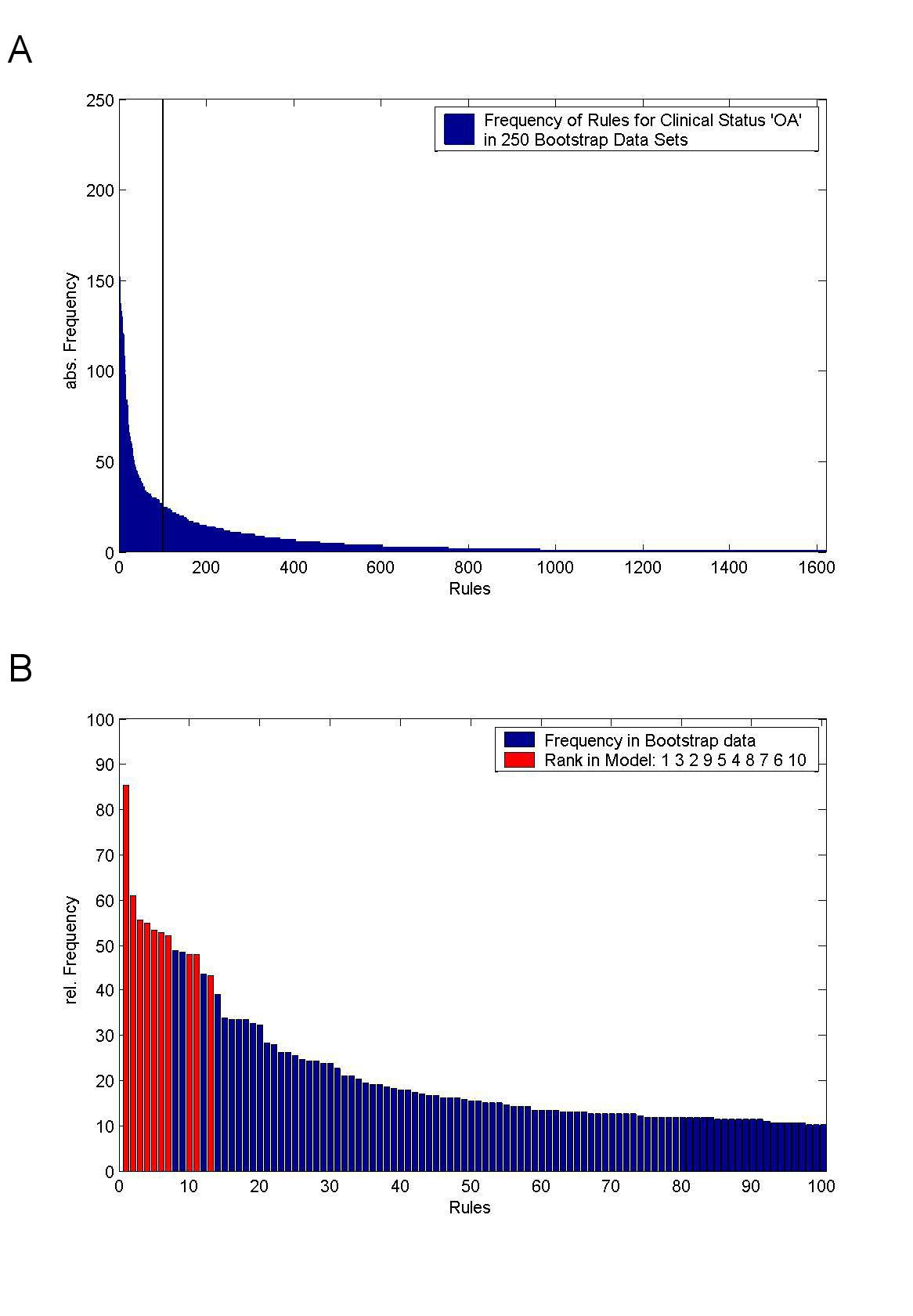


**Additional Figure A2.5**

Result of the internal validation of trained rule set for study group ‘Jena’ and conclusion ‘OA’. A: Absolute frequency of rules obtained from 250 Bootstrap Data Sets; B: Relative frequency of the 100 most frequent rules obtained from 250 Bootstrap Data Sets; red: the 10 rules of the optimized (pruned) rule set (with rank 1, 3, 2, 9, 5, 4, 8, 7, 6, 10).


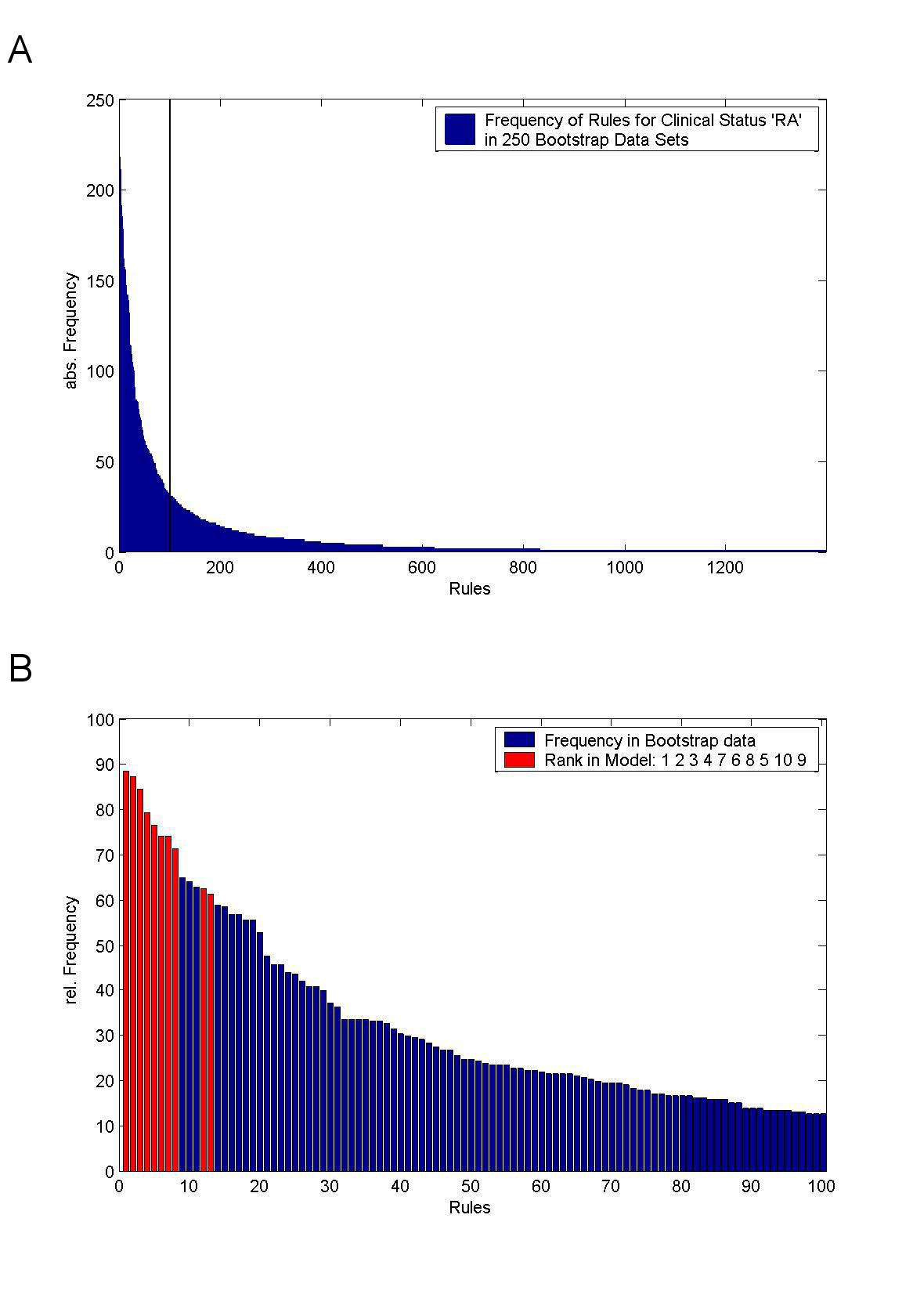


**Additional Figure A2.6**

Result of the internal validation of trained rule set for study group ‘Jena’ and conclusion ‘RA’. A: Absolute frequency of rules obtained from 250 Bootstrap Data Sets; B: Relative frequency of the 100 most frequent rules obtained from 250 Bootstrap Data Sets; red: the 10 rules of the optimized (pruned) rule set (with rank 1, 2, 3, 4, 7, 6, 8, 5, 10, 9).


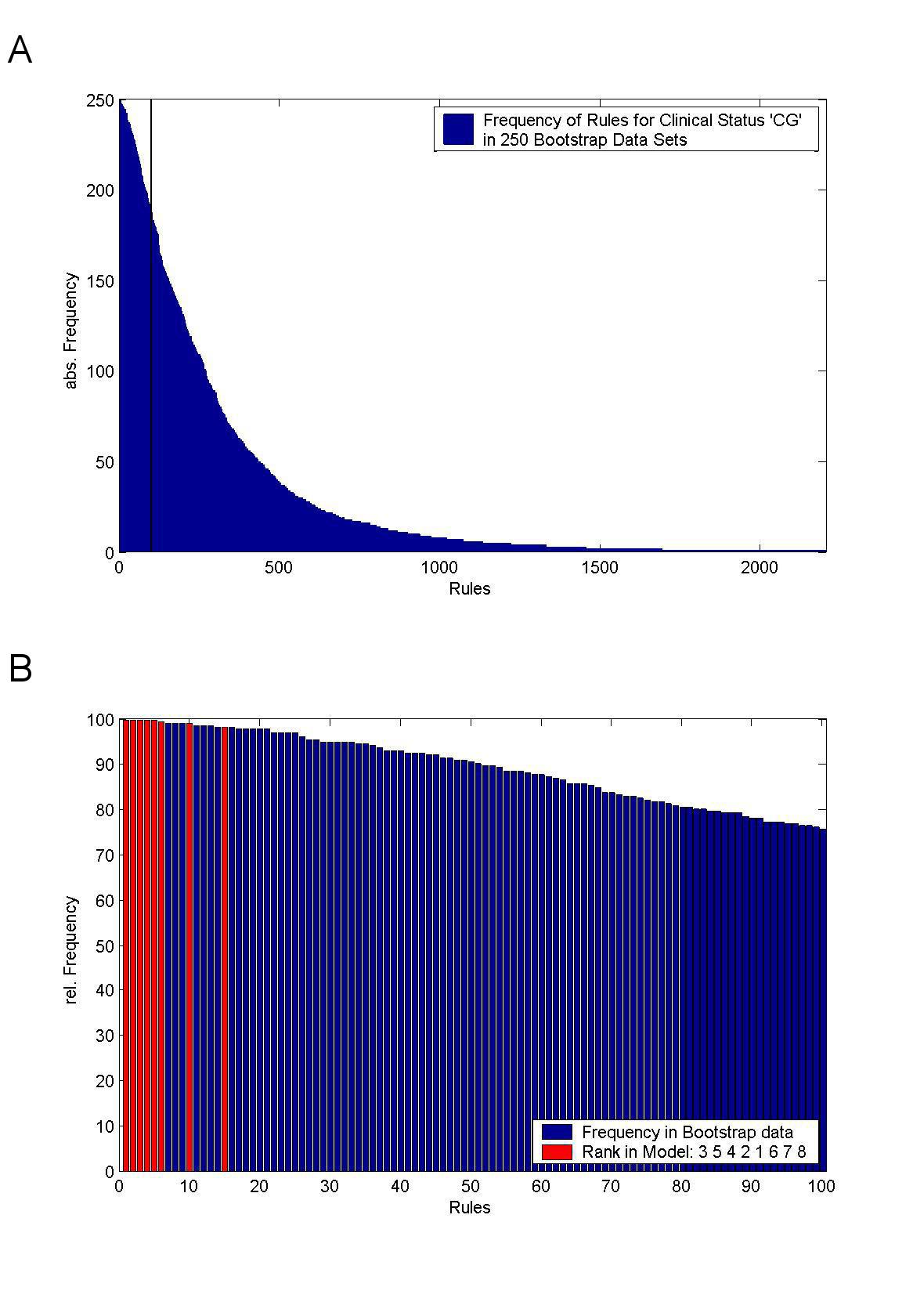


**Additional Figure A2.7**

Result of the internal validation of trained rule set for study group ‘Berlin’ and conclusion ‘CG’. A: Absolute frequency of rules obtained from 250 Bootstrap Data Sets; B: Relative frequency of the 100 most frequent rules obtained from 250 Bootstrap Data Sets; red: the 8 rules of the optimized (pruned) rule set (with rank 3, 5, 4, 2, 1, 6, 7, 8).


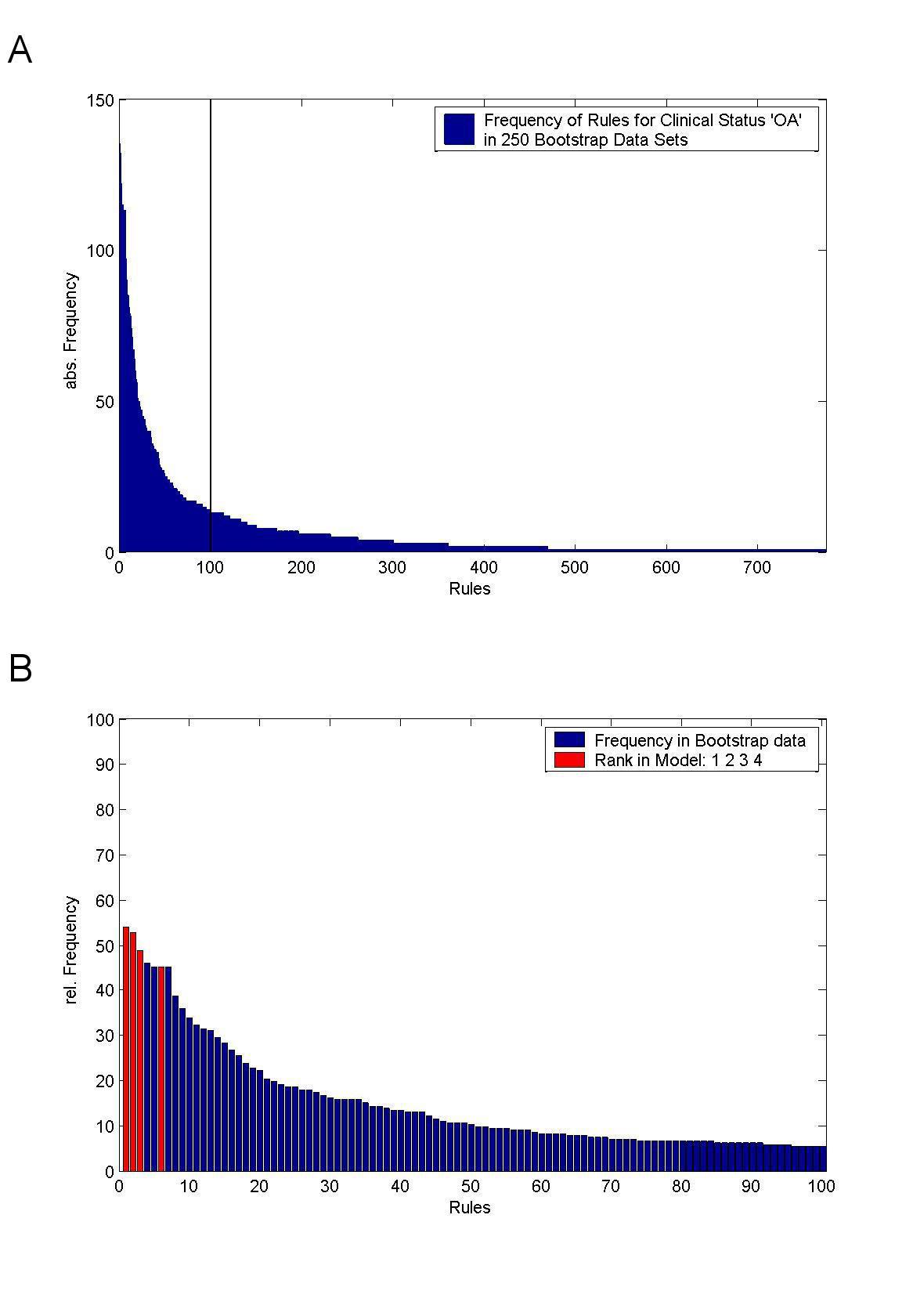


**Additional Figure A2.8**

Result of the internal validation of trained rule set for study group ‘Berlin’ and conclusion ‘OA’. A: Absolute frequency of rules obtained from 250 Bootstrap Data Sets; B: Relative frequency of the 100 most frequent rules obtained from 250 Bootstrap Data Sets; red: the 4 rules of the optimized (pruned) rule set (with rank 1, 2, 3, 4).


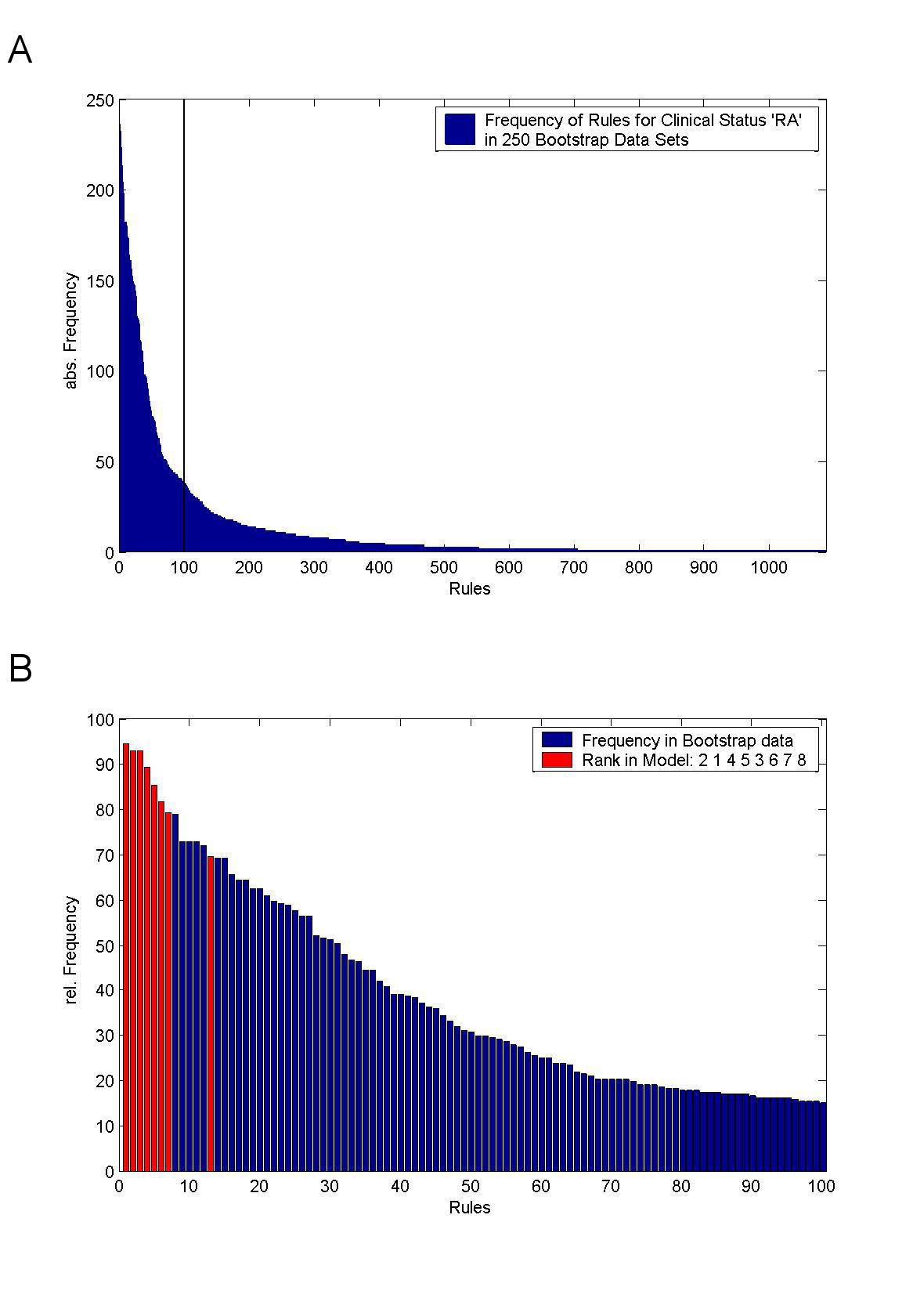


**Additional Figure A2.9**

Result of the internal validation of trained rule set for study group ‘Berlin’ and conclusion ‘RA’. A: Absolute frequency of rules obtained from 250 Bootstrap Data Sets; B: Relative frequency of the 100 most frequent rules obtained from 250 Bootstrap Data Sets; red: the 8 rules of the optimized (pruned) rule set (with rank 2, 1, 4, 5, 3, 6, 7, 8).


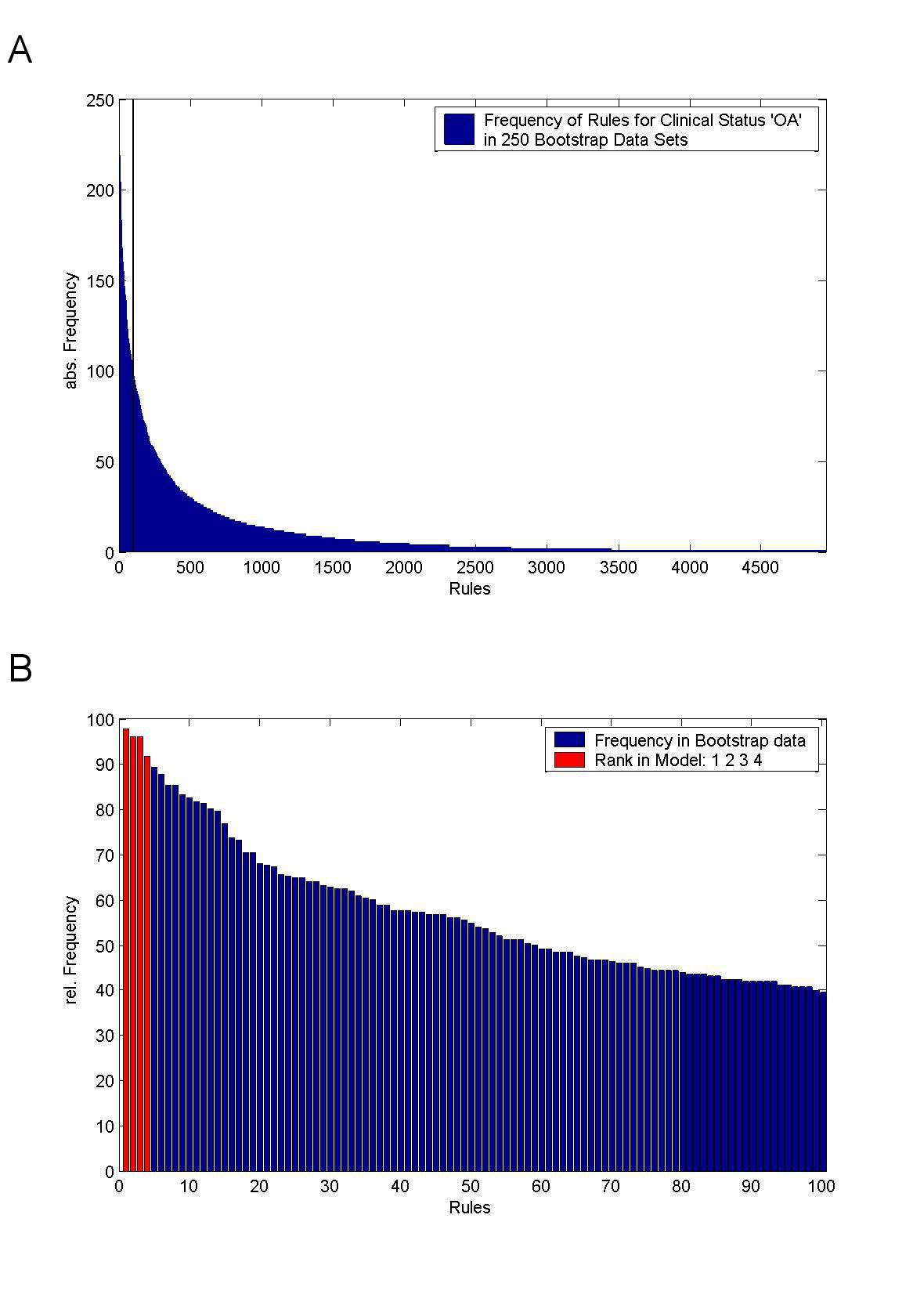


**Additional Figure A2.10**

Result of the internal validation of trained rule set for study group ‘Leipzig’ and conclusion ‘OA’. A: Absolute frequency of rules obtained from 250 Bootstrap Data Sets; B: Relative frequency of the 100 most frequent rules obtained from 250 Bootstrap Data Sets; red: the 4 rules of the optimized (pruned) rule set (with rank 1, 2, 3, 4).


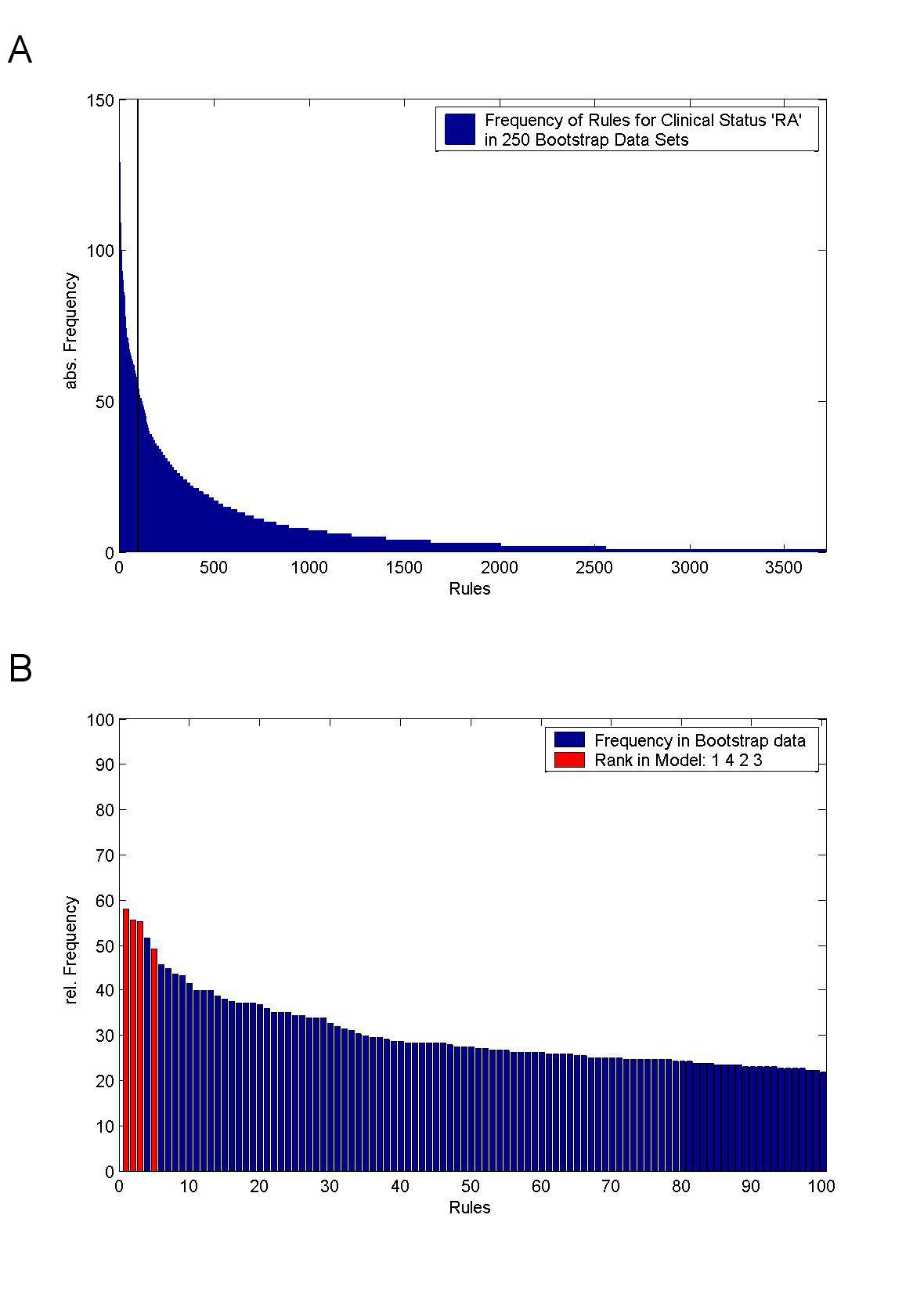


**Additional Figure A2.11**

Result of the internal validation of trained rule set for study group ‘Leipzig’ and conclusion ‘RA’. A: Absolute frequency of rules obtained from 250 Bootstrap Data Sets; B: Relative frequency of the 100 most frequent rules obtained from 250 Bootstrap Data Sets; red: the 4 rules of the optimized (pruned) rule set (with rank 1, 4, 2, 3).


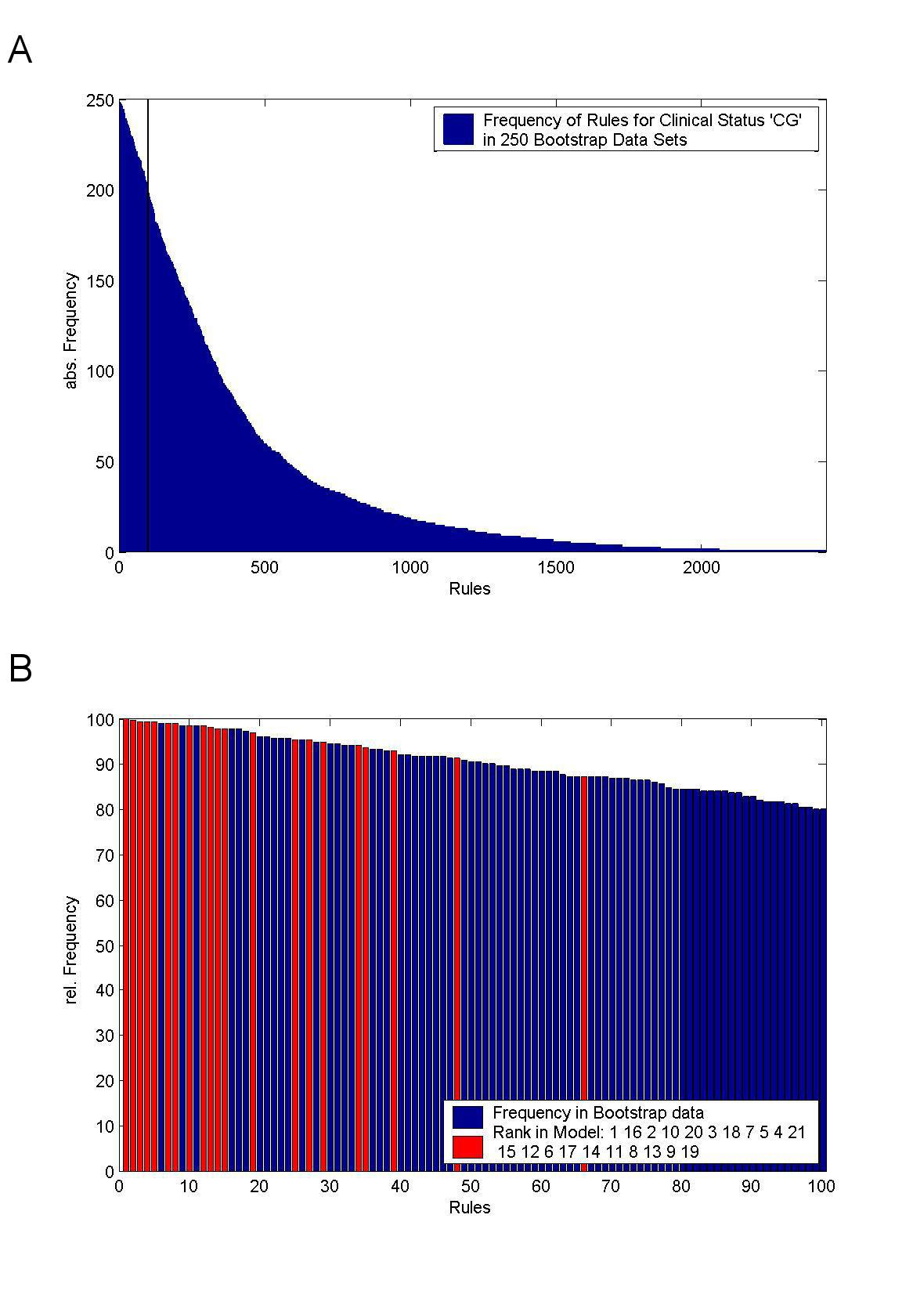


**Additional Figure A2.12**

Result of the internal validation of trained rule set for study group ‘Total’ and conclusion ‘CG’. A: Absolute frequency of rules obtained from 250 Bootstrap Data Sets; B: Relative frequency of the 100 most frequent rules obtained from 250 Bootstrap Data Sets; red: the 21 rules of the optimized (pruned) rule set (with rank 1, 16, 2, 10, 20, 3, 18, 7, 5, 4, 21, 15, 12, 6, 17, 14, 11, , 8, 13, 9, 19).


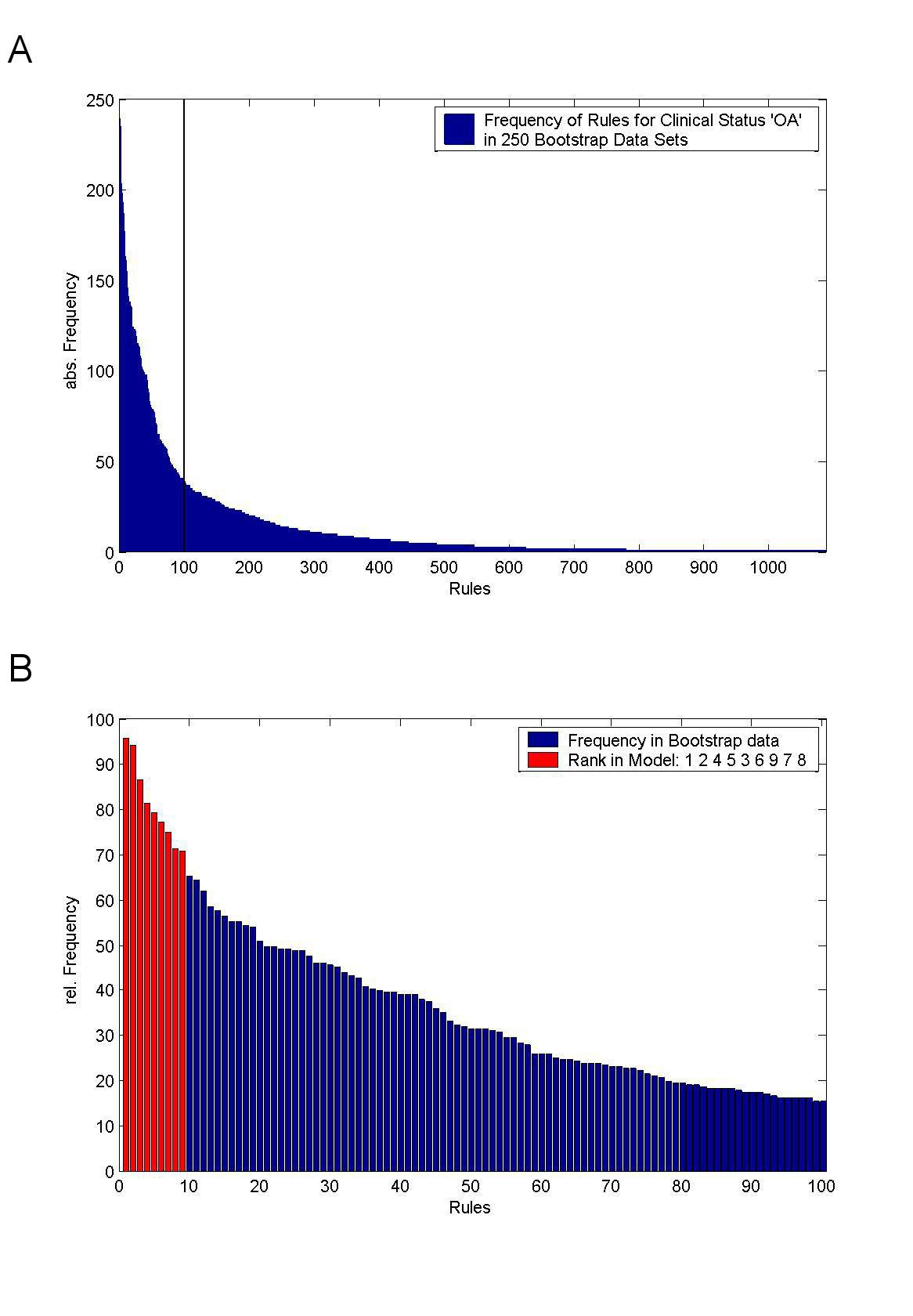


**Additional Figure A2.13**

Result of the internal validation of trained rule set for study group ‘Total’ and conclusion ‘OA’. A: Absolute frequency of rules obtained from 250 Bootstrap Data Sets; B: Relative frequency of the 100 most frequent rules obtained from 250 Bootstrap Data Sets; red: the 9 rules of the optimized (pruned) rule set (with rank 1, 2, 4, 5, 3, 6, 9, 7, 8).


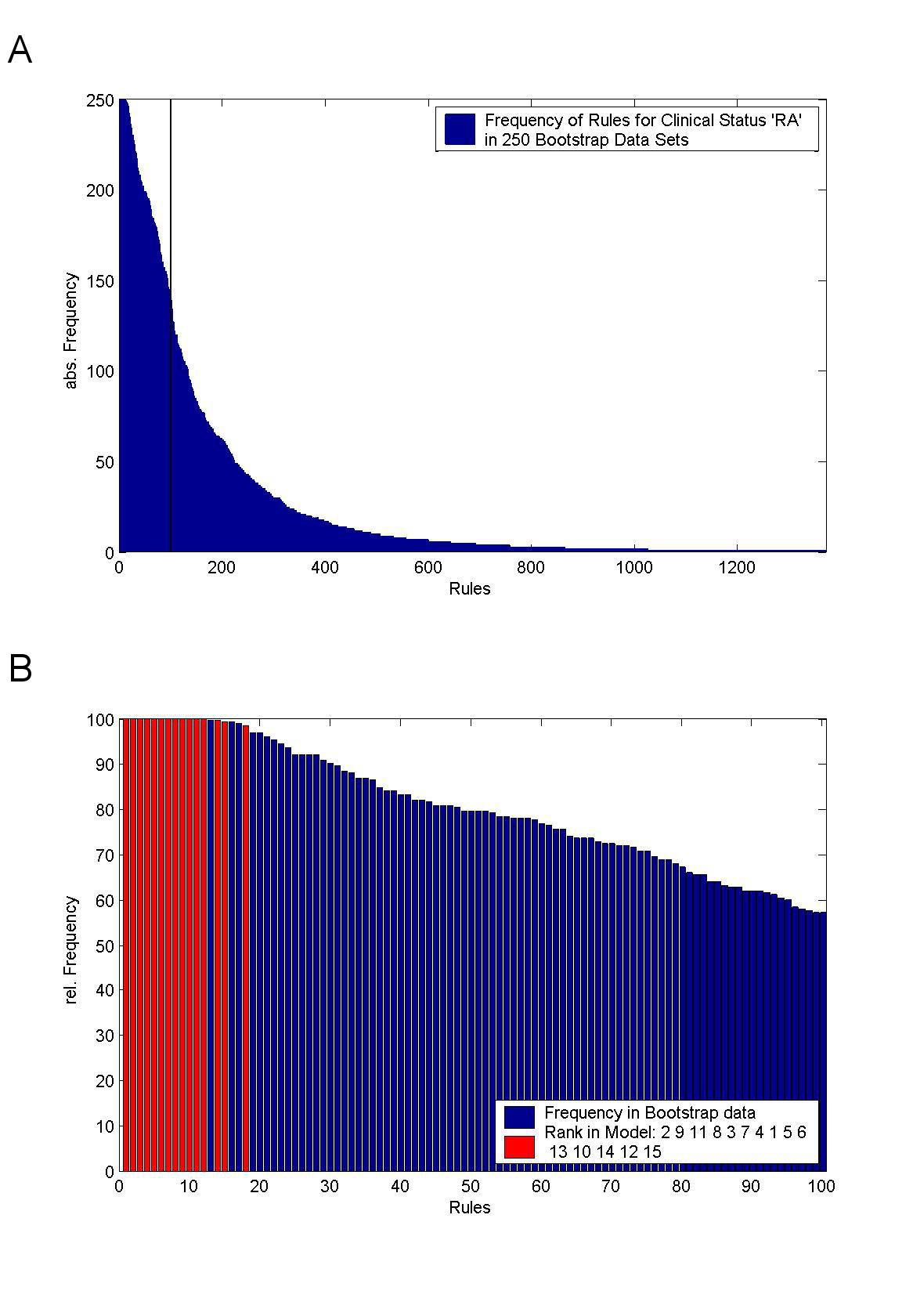


**Additional Figure A2.14**

Result of the internal validation of trained rule set for study group ‘Total’ and conclusion ‘RA’. A: Absolute frequency of rules obtained from 250 Bootstrap Data Sets; B: Relative frequency of the 100 most frequent rules obtained from 250 Bootstrap Data Sets; red: the 15 rules of the optimized (pruned) rule set (with rank 2, 9, 11, 8, 3, 7, 4, 1, 5, 6, 13, 10, 14, 12, 15).
